# Zinc-nanoparticles alleviate the ovarian damage induced by bacterial lipopolysaccharide (LPS) in pregnant rats and their fetuses

**DOI:** 10.1007/s00418-023-02222-4

**Published:** 2023-07-26

**Authors:** Abd El-Fattah B. M. El-Beltagy, Samaa M. Bakr, Samah S. G. Mekhaimer, Noura F. Ghanem, Amany Attaallah

**Affiliations:** 1 Zoology Department, Faculty of Science, Damanhour University, Damanhour, Egypt; 2 Zoology Department, Faculty of Science, Kafrelsheikh University, Kafrelsheikh, Egypt

**Keywords:** LPS, Gestation, Pups, Ovary, Zn-NPs, Apoptosis, Antioxidants

## Abstract

Lipopolysaccharide (LPS) is an endotoxin derived from the cell wall of Gram-negative bacteria. LPS exposure during early gestation is associated with adverse effects on the placenta as well as on developmental outcomes, including embryonic resorption, fetal death, congenital teratogenesis, and fetal growth retardation. This work aimed to explore the adverse effects of LPS injected at an early stage of gestation on the gonads of pregnant rats and the ovaries of their pups and the role of zinc nanoparticles (Zn-NPs) against these adverse effects. Twenty-four pregnant rats were used in this study. They were divided at gestation day 4 into four groups (*n* = 6): control, Zn-NPs (20 mg/kg orally from gestation day E14 till the end of weaning), LPS (50 µg/kg at gestation days E7 and E9), and LPS + Zn-NPs group. The body weight and placenta weight were recorded at gestational day 16. At postnatal day 21 (weaning), the mothers rats and their offspring were sacrificed and immediately dissected to remove the ovaries and uteri from the mothers and the ovaries from their offspring for subsequent biochemical, histological, and immunohistochemical investigations. The obtained results revealed that LPS exposure during early gestation caused severe histopathological alterations in the placenta, uterus, and ovaries of mothers, as well as in the ovaries of their pups. Also, the uterine and ovarian sections displayed a positive reaction for caspase-3 antibody and a negative reaction for Bcl-2 antibody, which reflects the apoptotic effect of LPS. Additionally, remarkable reductions in the levels of antioxidants (superoxide dismutase and catalase) and significant increases in malondialdehyde (MDA) levels were recorded in the serum of LPS-treated mothers and in the ovarian tissues of their offspring. Further biochemical analysis of the ovarian tissues from LPS-maternally treated offspring showed a significant increase in the levels of caspase-3, TNF-α, and TGF-β1, but a significant decrease in the level of IGF-1. On the other hand, treatment of mothers with Zn-NPs from day 14 of gestation until the weaning day (21st day postnatal) successfully ameliorated most of the deleterious histopathological, immunohistochemical, and biochemical changes induced by LPS.

**Graphical abstract:**

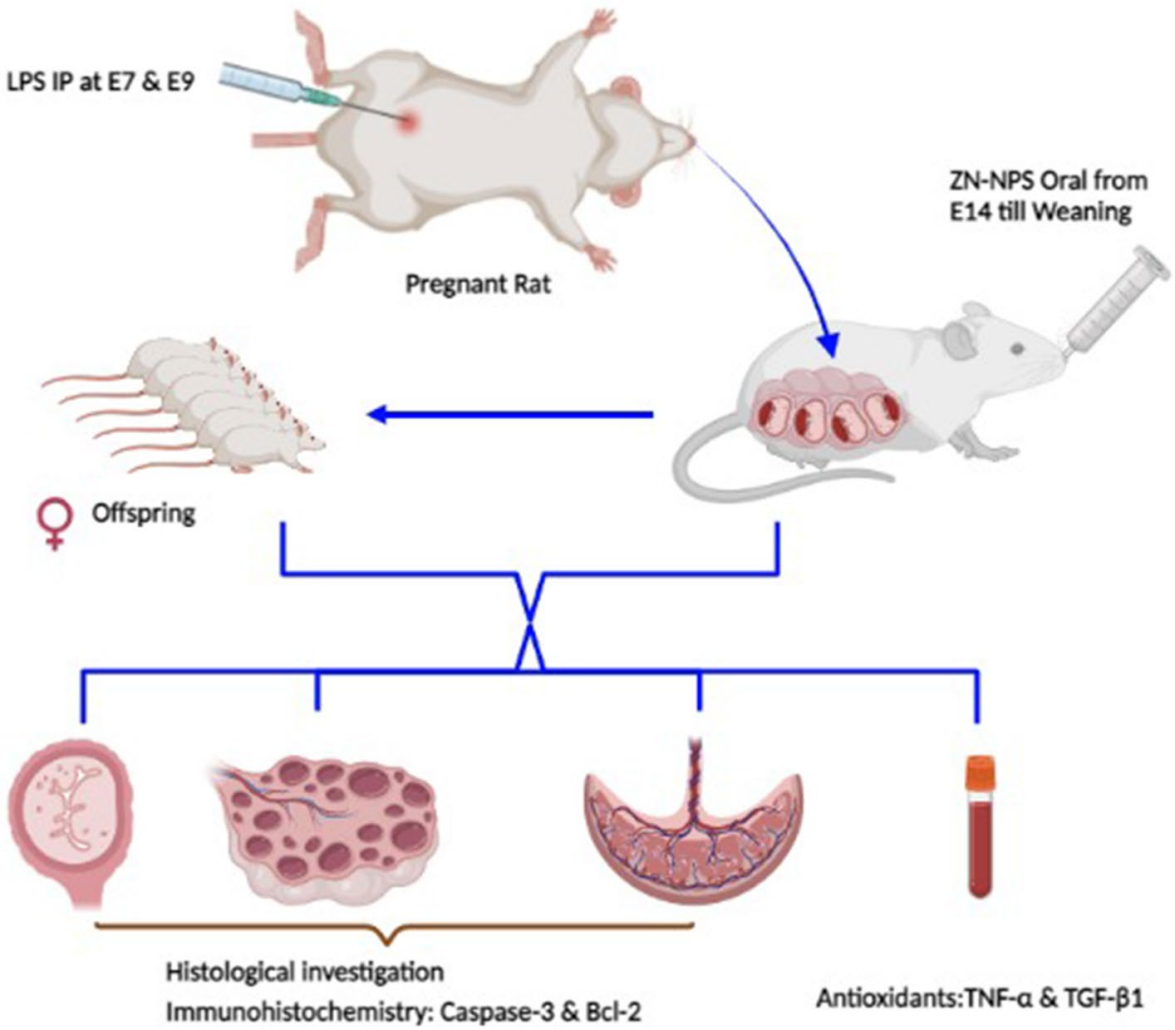

## Introduction

Maternal microbial infections are one of the main causes of preterm labor and adverse fetal outcomes, which persist as major and unresolved challenges. Lipopolysaccharide (LPS) is a toxic component of the cell walls of most Gram-negative bacteria (Jacob et al. 1977). Systemic maternal LPS exposure has been previously shown to be associated with adverse fetal developmental outcomes such as embryonic resorption, intrauterine fetal death, and preterm labor in animals (O’Sullivan et al. 1988). Exposure of pregnant rats to bacterial LPS resulted in maternal inflammations and fetal loss, as well as structural abnormalities in the uteroplacental vasculature, decreased placental blood flow, and placental and fetal hypoxia within 3 h of LPS administration (Renaud et al. 2011). Intraperitoneal injection of LPS in rats during gestation increased the levels of placental inflammatory cytokines and placental pathological damage, decreased the levels of vascular endothelial growth factor (VEGF), reduced the number of live fetuses, and induced fetal growth factor restriction (Bao et al. 2019). Also, intraperitoneal injection of a low dose of LPS (0.5 mg/kg) once daily for 6 days markedly reduces the sizes of the ovaries and uteri of mice through activation of proinflammatory mediators like interleukin 1β (IL-1β), and IL-6 in the mouse ovaries. These mediators can induce inflammation, granulosa cell death, and fibrosis in mouse ovaries, resulting in primary ovarian insufficiency (Wang et al. 2020; Ning et al. 2008). Other studies revealed that binding of LPS to macrophages activates synthesis and releases tumour necrosis factor (TNF-α) and nuclear factor kappa-B (NF-κB) in the placenta and fetal liver (Urakubo et al. 2001). Yu et al. reported that maternal exposure to LPS (0.79 mg/kg) produced a significant increase in serum and hepatic levels of total cholesterol, triglycerides, low-density lipoprotein cholesterol, aspartate amino transferase, and liver morphological abnormalities in 8-week-old offspring rats (Yu et al. 2018).

Zinc (Zn) is a vital trace element found in all tissues and body fluids. It plays a crucial role in cellular signaling (Oteiza and Mackenzie 2005). Zinc regulates more than 300 metalloenzymes and plays an important role in signal transduction, cellular proliferation, apoptosis, gene expression, and infant neurological development (Nissensohn et al. 2016). Zn is important in normal reproduction and fetal development (Bailey et al. 2015). Zn is essential for both male and female reproductive potential, as it is necessary for normal fertilization. Deficiency of Zn results in impairment of reproductive function in both genders (Nadjarzadeh et al. 2013). Zn is essential as an antioxidant and antiinflammatory (Choi et al. 2018). The highest risk factor for disease mortality and morbidity, with nearly 20% of perinatal mortalities on the global level, is zinc deficiency (Nriagu 2011). Zn is also necessary for maintaining follicle-stimulating hormone (FSH) and luteinizing hormone (LH) levels (Barsony et al. 2013) and consequently normal ovarian functions (Yao et al. 2023). The mechanism of Zn metabolism in both males and females is based on interactions between Zn and sexual hormone receptors (Hojyo and Fukada 2016). In the absence or reduction of Zn metalloenzyme, sex hormones in both males and females cannot be activated (Prasad 2013). Zn deficiency is associated with significant activation of caspase-3 (an apoptotic marker) and downregulation of Bcl-2 (an antiapoptotic marker), which results in apoptosis (Thomas et al. 2013).

Zn plays a major role in maternal, infant, and neonatal survival (Deshpande et al. 2013). Zn deficiency during gestation is associated with a lower birth weight, and a high Zn deficiency can lead to spontaneous abortion and disturbance in the development of embryos (Jyotsna et al. 2015). Zn deficiency in the mother can be inherited by the infant. These infants may display symptoms such as alopecia, appetite loss, diarrhea, impaired immune-related functions, and dermatitis (Nenkova et al. 2017).

Absorption of zinc takes place in the gastrointestinal tract (GIT) in very low amounts and differs with the age of the animal (Zhao et al. 2014). Thus, it is better to find a new approach to prevent Zn loss from the body as well as use a low dose of Zn if possible. Negahdary reported that zinc nanoparticles (Zn-NPs) have better antibacterial effects than traditional zinc, especially against Gram-positive and Gram-negative bacteria (Negahdary 2012). Zn-NPs are being used in the food industry as additives and during packaging to resist microbial infection (Gerloff et al. 2009; Jin et al. 2009). Studies have already proven the dose-dependent effect of Zn-NP on growth performance in livestock and poultry (Lina et al. 2009; Mishra et al. 2014) and as an antimicrobial and immune-modulatory agent by reducing the diarrhea rate in piglets. Zn-NPs have been documented to improve growth performance, enhance feed utility, and provide economic benefits in weaning piglets and poultry (Mishra et al. 2014).

Zn-NPs have antibacterial effects on both Gram-positive and Gram-negative bacteria. The antibacterial activity of Zn-NP depends on the surface area and concentration, whereas the crystalline form has little effect while the powder nano-form has the highest effects (Negahdary 2012). The authors added that Zn-NP carries a positive charge while the microorganisms carry a negative charge, creating an “electromagnetic” attraction between the microbe and the treated surface. This attraction helps facilitate the penetration of Zn-NPs into the bacterial cell and their interaction with phosphorus- and sulphur-containing compounds like DNA, resulting in cell damage. Furthermore, Zn-NPs also resist bacterial adhesion to the cell membranes (Padmavathy and Vijayaraghavan 2008).

According to the above-mentioned adverse effects of LPS from bacterial cell wall and the beneficial role of zinc or its nanoparticles during the gestation period, this work aims to evaluate the ameliorative role of zinc nanoparticles against ovarian dysfunction in LPS-treated pregnant rats and their pups.

## Materials and methods

### Chemicals

Lipopolysaccharide (LPS) (*Escherichia coli*, serotype 055:B5) and Zn-NPs were purchased from Sigma-Aldrich Co. LLC. GmbH, Steinheim, Germany Size. Zn-NPs was in the form of nanopowder at 35 nm, and its surface area was 40 m^2^/g.

### Induction with LPS

A total of 1.5 mg of LPS was dissolved in 30 mL distilled water. Pregnant rats were injected interaperitoneally with two doses of LPS (150 µg/kg) on gestation days E7and E9 (Wang et al. 2014).

### Preparation of Zn nanoparticle suspension

Zn nanopowder was suspended directly in deionized water at concentrations of 2000 μg /L then dispersed using ultrasonic vibration (40 kHz) for 30 min to prevent NP aggregation. Zn-NPs shape and size were evaluated using the transmission electron microscope (TEM) (JEM-1011, JEOL, Japan).

### Treatment with Zn-NPs

The pregnant rats daily received Zn-NPs (20 mg/kg via gavage) from the gestation day E14 till the end of weaning (Jafari et al. 2017).

### Experimental animals

For this study, 32 Wistar albino rats (24 females and 8 males) weighing 150 ± g were obtained from the Holding Company for Biological Products and Vaccines (VACSERA, Cairo, Egypt). The animals were kept in animal houses under standard condition of illumination with a 12-h light–dark cycle at 25 ± 1 °C and 50% relative humidity. They were provided with tap water and a balanced diet ad libitum. After an acclimatization period of 1 week, the animals were mated in the special matting cages (1 male: 3 females) overnight. After 3–4 days and ensuring pregnancy via observation of vaginal plug and using vaginal smear method, pregnant females were separated from males. The pregnant rats were divided into four groups (six for each group): group 1 (control)—pregnant rats were fed on usual food and distilled water, group 2 (Zn-NPs)—they were treated with Zn-NPs (20 mg/kg) from gestation day E14 till the end of weaning, group 3 (LPS)—the pregnant rats were injected with intraperitoneal (IP) dose of LPS (50 µg/kg) at gestation days E7and E9 (Wang et al. 2014), and group 4 (LPS and Zn-NPs)—the pregnant rats were injected with LPS at gestation days E7and E9 followed by Zn-NPs from gestation day 14 until weaning by the same previous doses in groups 2 and 3.

### Sample collection and tissue preparation

On day 16 of gestation, three pregnant female rats from each group were weighed and dissected to investigate the weight and histopathological changes of the placenta. On postnatal day 21 (the end of the experiment), the other three mother rats for each group were sacrificed by decapitation, and the blood samples were withdrawn and collected in glass tubes. The serum was separated by centrifugation at 3000 rpm for 10 min and stored at –80 °C for biochemical analysis. The mother rats were dissected, and the whole ovaries and uterus were removed, while their offspring were dissected to remove the ovaries. The ovaries and uteri of mother rats were processed for histological and immunohistochemical investigations, while the ovaries of offspring were processed for subsequent histological, immunohistochemical, and biochemical investigations. All procedures were performed in accordance with the guidelines of the bioethics committee of Damanhour University, which approved the animal experiments.

### Investigated parameters

#### Body weight

Mother rats were weighed on the 16th day of gestation and at the end of weaning.

#### Histological investigation

Half of the obtained organs (uterus, placenta, and ovaries of mothers and the ovaries of offspring) were washed in normal saline and fixed in 10% neutral buffered formalin. After fixation, the specimens were dehydrated with an ascending ethanol series, cleared with xylene, and embedded in paraffin. The sections were stained with Mayer’s hematoxylin and eosin (H&E) (Bancroft and Gamble 2008). The sections were investigated under a bright field light microscope (Olympus^®^) and photographed by an Olympus^®^ digital camera installed on the microscope to investigate the histological and histopathological signs of gonads.

#### Immunohistochemical staining technique

##### Immunohistochemical labeling of Bcl-2

The paraffin-embedded sections of uterus and ovaries from mothers and ovaries from their pups were blocked by goat serum for 20 min at 37 °C to inhibit non-specific antibody binding and then incubated separately with primary antibodies (1:100 dilution; Santa Cruz Biotechnology, Santa Cruz, CA) (mouse anti-human Bcl-2) at 4 °C overnight. After being washed three times in PBS (3 min each time), the sections were incubated in biotin-labelled goat anti-mouse IgG (Alexa Fluor^®^ 488) at 37 °C for 30 min. The sections were washed again with PBS, followed by incubation with the streptavidin peroxidase complex for 30 min at 37 °C. Staining was visualized with 3,3′-diaminobenzidine (DAB) for 10 min at room temperature.

##### Immunohistochemical labeling of caspase-3

The 5-μm thick paraffin-embedded sections of the uterus and ovaries from mother rats and the ovaries from their pups were cut, mounted onto positively charged slides, deparaffinized, rehydrated in descending grades of alcohol, and washed in PBS. Endogenous peroxidase activity was inhibited using 3% H_2_O_2_ in methanol for 40 min at room temperature. The tissue sections were retained at normal room temperature and processed for antigen retrieval by digestion with 0.05% trypsin. After thorough washing in TRIS buffered saline (TBS), pH 7.6, the sections were incubated for 45 min with diluted 1:10 monoclonal primary antibody (anti-caspase-3 antibody, clone 3J16, ZooMAb^®^ rabbit monoclonal). To detect the specificity of this antibody, anti-caspase-3 antibody was incubated with the peptide used to generate the antibody (cell signaling; antibody/peptide 1:1), and anti-caspase-3 antibody was incubated with recombinant caspase-3 (Calbiochem, San Diego, CA; antibody/recombinant protein 1:5). Slides were then rinsed in PBS and subsequently incubated in the presence of the secondary antibody biotinylated anti-rabbit IgG (Amersham Pharmacia Biotech, Bucks) for 20 min. For all sections, the complex sites were shown brown using 3,3-diaminobenzidine tetrahydrochloride with fresh hydrogen peroxide substrate (Rohan et al. 1998).

The sections were counterstained with Mayer’s hematoxylin, mounted, and photographed by phase contrast light microscopy (with an Olympus^®^ digital camera installed on an Olympus^®^ microscope). The incidences of cellular accumulations of Bcl-2 and caspase-3 proteins were determined for each group. Additionally, the images were analyzed on an Intel^®^ Core I7^®^-based computer using Video Test Morphology^®^ software (Russia) with a specific built-in routine for area, % area, measurement, object counting, and contact angle.

#### Serum analysis in mother rats

##### Measurement of antioxidants (catalase and superoxide dismutase) and malondialdehyde (MDA)

The level of catalase (CAT) was determined in serum using a previous method (Koroliuk et al. 1988). Briefly, 10  μL of serum sample was incubated with 100 μmol/mL of H_2_O_2_ in 0.05 mmol/L Tris–HCl buffer, pH 7, for 10 min. The reaction was terminated by rapidly adding 50  μL of 4% ammonium molybdate. The yellow complex of ammonium molybdate and H_2_O_2_ was measured at 410 nm. One unit of catalase activity was defined as the amount of enzyme required to decompose 1 μmol H_2_O_2_ per minute.

The determination of the superoxide dismutase (SOD) level in serum was based on the method of Bahrami et al. (2016). Briefly, 300  μL of the mixed substrate was added to 200  μL of diluted hemolysates. The samples were mixed well, and 75 μL xanthine oxidase was added to the reactions. The absorbance was measured at 505 nm, and the SOD activity was then calculated according to the manufacturer’s instructions (Ransod^®^-Randox Lab, Antrim, UK).

The thiobarbituric acid reaction method of Placer et al. was used to measure the malondialdehyde (MDA) levels in serum (Placer et al. 1966). Quantification of the thiobarbituric acid-reactive substances was determined at 532 nm by comparing the absorption to the standard curve of MDA equivalents generated by acid-catalyzed hydrolysis of 1,1,3,3-tetramethoxypropane. To measure the MDA level, a working solution containing 15% trichloroacetic acid, 0.375% thiobarbituric acid, and 0.25 N hydrochloric acid was prepared. For each sample, 250  μL serum and 500  μL working solution were mixed and placed in boiling water for 10 min. After cooling, the samples were centrifuged at 3000 rpm for 10 min. Finally, 200  μL of each supernatant was transferred to microplates, and the optical density of the samples was measured at 535 nm.

##### Measurement of follicular stimulating hormone (FSH)

Serum FSH was determined with ELISA using specific diagnostic kits (DRG Instruments GmbH, Germany). To obtain accurate results from hormonal assays, we measured the level of FSH in the follicular phase according to the sex cycle of each rat (Westwood 2008).

#### Biochemical analysis in the ovarian tissues of 21 days old offspring

##### Preparation of ovarian tissues homogenate

The ovarian tissues were homogenized (100 mg wet weight/mL) in PBS (10 mM sodium phosphate and 150 mM sodium chloride, pH 7.8) containing 0.2% Triton X-100 with a Teflon glass tissue grinder on ice (15 strokes). Homogenates were centrifuged (3000*g* at 4 °C for 15 min), and the supernatant fractions were collected, stored at −70 °C, and later used for enzyme-linked immunosorbent assay (ELISA).

##### Estimation of ovarian tissues antioxidants (CAT and SOD) and MDA

Determination of CAT activity was based on the method of the previous protocol by Sinha (Sinha 1972). Briefly, the assay mixture containing 1 mL of PBS (pH 7), 100 µL of ovarian tissue lysate, and 400 µL of 2 M hydrogen peroxide (H_2_O_2_) was incubated for 1 min, and the reaction was stopped by the addition of 2.0 mL of dichromate acetic acid reagent (5% potassium dichromate and glacial acetic acid in a ratio of 1:3). Using a spectrophotometer (Eppendorf Bio-Photometer), the absorbance was measured at 570 nm. The activity was expressed as µM of H_2_O_2_ consumed per minute/mg of protein (H_2_O_2_ consumed per minute/mg protein). A mixture devoid of tissue homogenate was taken as a blank.

The analytical protocol of Kakkar et al. was used to determine the superoxide dismutase (SOD) activity (Kakkar et al. 1984). Briefly, a mixture containing 50 µL of 10% ovarian tissue homogenate, 600 µL of 52 mM sodium pyrophosphate buffer (pH 8.3), 50 µL of 186 µM phenazine methosulfate (PMS), and 150 µL of 300 µM nitroblue tetrazolium (NBT). The reaction was started by the addition of 100 µL of 750 µM ml NADH and incubated at 30 °C. The reaction was terminated after 90 s incubation by the addition of 500 µL glacial acetic acid. A total of 2 mL of n-butanol were added, vortexed, and allowed to stand for 10 min. The mixture was centrifuged at 10,000*g* for 10 min at room temperature, and the supernatant was collected. Color intensity was measured at 560 nm.

Malondialdehyde (MDA) levels in the ovarian lysate were determined according to the principle previously described by Ohkawa et al. (1979). In brief, 75 µL of the lysates were mixed with the same amount of 10% trichloroacetic acid (TCA). Freshly prepared 0.2% thiobarbituric acid was then added in a ratio of 1:2 and then boiled for 45 min. The solutions were allowed to cool down, and the optical densities were measured against the blank at 532 nm and the concentration of MDA was expressed as nmol/mL.

##### Measurement of caspase-3 in ovarian tissues of 21 day old offspring

In a 100-unit volume of ovarian tissue lysate, a 10-unit buffer or sample was incubated with the substrate Ac-DEVD-qNA for 4 h at 37 °C. The absorbance was measured at 405 nm. The caspase-3 activity was expressed as a percentage of the control. All the detailed procedures were carried out according to the manufacturer’s protocols (Mi et al. 2013).

##### Measurement of insulin like growth factor-1(IGF-1) in ovarian tissues

IGF-1 level was determined in the ovarian homogenates using a double-antibody sandwich enzyme linked-immunosorbent assay (ELISA) kit (Elabscience, USA) (cat. no. E-EL-R0010, lot: E3YXQMELZQ).

##### Measurement of tumor necrosis factor-alpha (TNF-α) in the ovarian tissues

TNF-α was measured in the ovarian tissue homogenate by means of ELISA with specific monoclonal antibodies against these cytokines, using commercially available kits Quantikine and Quantikine HS from R and D Systems Europe, Ltd. The sensitivity thresholds of Quantikine and Quantikine HS assays are 0.038–0.191 pg/10 mg.

##### Measurement of transforming growth factor beta-1(TGF-β1) in ovarian tissues

The level of TGF-β1 was measured in ovarian lysate using a commercially available TGF-β1 ELISA kit (R and D Systems, Minneapolis, MN). The ovarian cells were transfected with a lentivirus vector specific for TGF-β1 or control and were seeded into six-well plates with 106 cells per well at 37 °C in 2 mL DMEM containing 2% FBS. The supernatants were assayed for TGF-β1 following the manufacturer’s instructions. Results were expressed as pg/mL after subtracting the amount of TGF-β1 in medium alone.

### Statistical analysis

Data were analyzed using IBM SPSS software package version 20.0. (Armonk, NY: IBM Corp). The Kolmogorov–Smirnov test was used to verify the normality of distribution. Quantitative data were described using mean, standard deviation (SD), median, and range. Significance of the obtained results was judged at the 5% level.

The used tests were: 1—*F*-test (ANOVA) for normally distributed quantitative variables, to compare between more than two groups, and post hoc test (Tukey) for pairwise comparisons.

## Results

### Changes in the body weights of mother rats and their placenta

**Fig. 1:**
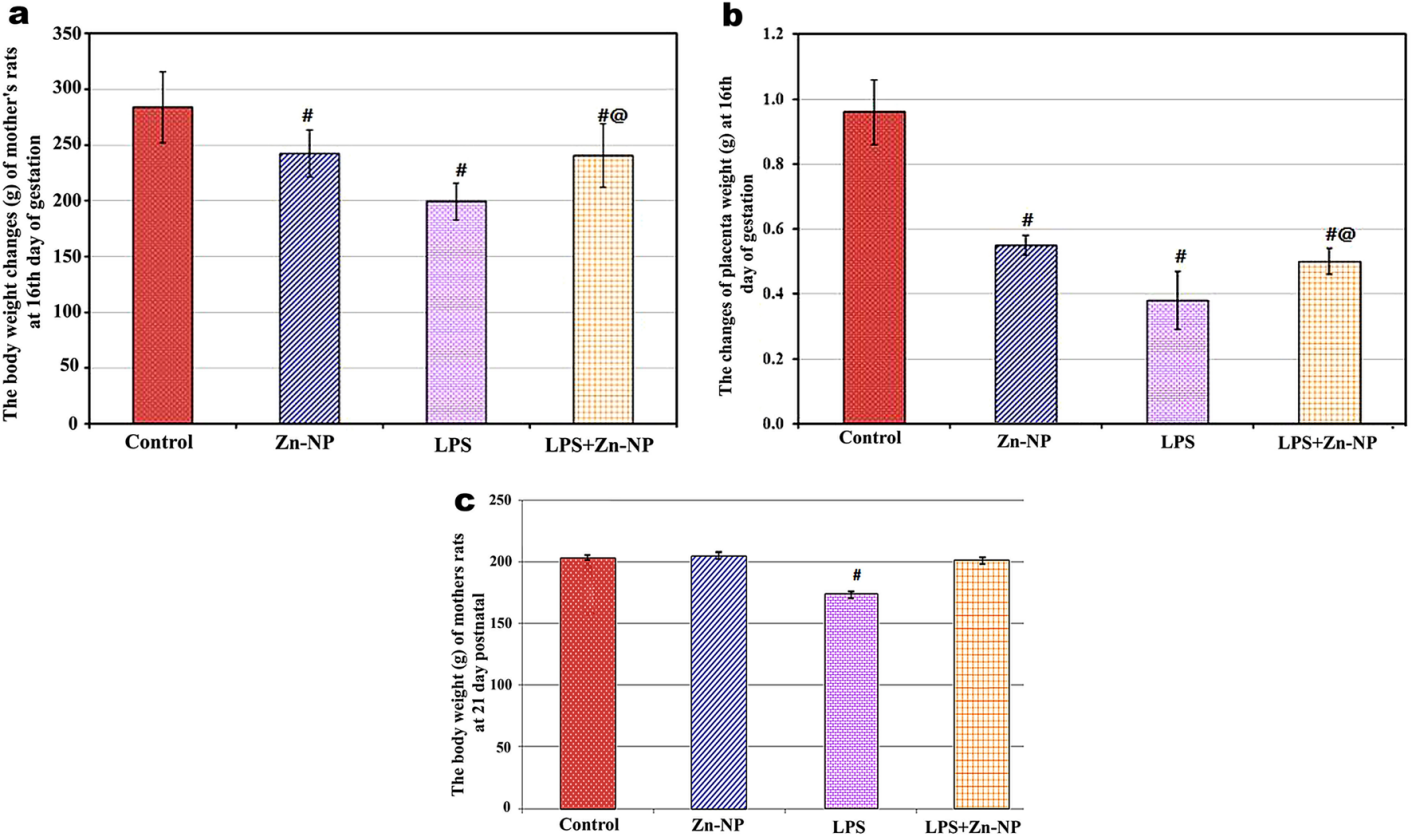
The body weight and placenta (not uterine) weight changes in pregnant rats at 16th day of gestation (panels **a** and **b**), and body weight changes at PND21 (panel **c**). Data was expressed in mean ± SD. *P* value for the comparison between the four studied groups *P*_1_: *P* value for comparing between LPS and LPS + Zn-NP, *Statistically significant at *P* ≤ 0.05. ^**#**^Significant with control, ^@^significant LPS with LPS + Zn-NP group in graph

Zn-NP-supplemented mother rats at the 16th day of gestation showed a significant decrease in their mean body weights (242.3 g ± 21.07 g) when compared with control (283.8 g ± 31.56 g); however, in LPS-treated mother rats, the body weight appeared significantly lowered (199.3 g ± 16.48 g) (*P* < 0.001) when compared with control. On the other hand, a highly significant increase in the mean body weight (240.8 g ± 28.42 g) was recorded in LPS cosupplemented with Zn-NP pregnant rats (at the 16th day of gestation) when compared to the LPS group alone (*P* < 0.001), but was still significantly lower when compared with the control (Fig. 1a).

The mean weight of the placenta at the 16th gestational day appeared significantly lower in Zn-NP (0.55 g ± 0.03 g), LPS (0.38 g ± 0.09 g), and LPS + Zn-NP (0.50 g ± 0.04 g) treated mother rats when compared with control (0.96 g ± 0.10 g). But, as seen from these results, the significant decrease in the placenta weight appeared more prominent in the LPS-treated group (*P* < 0.001) (Fig. 1b).

At the 21st day postnatal, the mother rats showed no significant change in their body weight between the Zn-NP-supplemented group and the control; however, a highly significant decrease in body weight was noticed in the LPS-treated mother rats when compared with the control. In LPS-exposed mother rats post-treated with Zn-NP, the body weight appeared to show a non-significant change compared to control (Fig. 1c).

### Histopathological observations in the ovaries, uterus, and placenta of mother rats

**Fig. 2:**
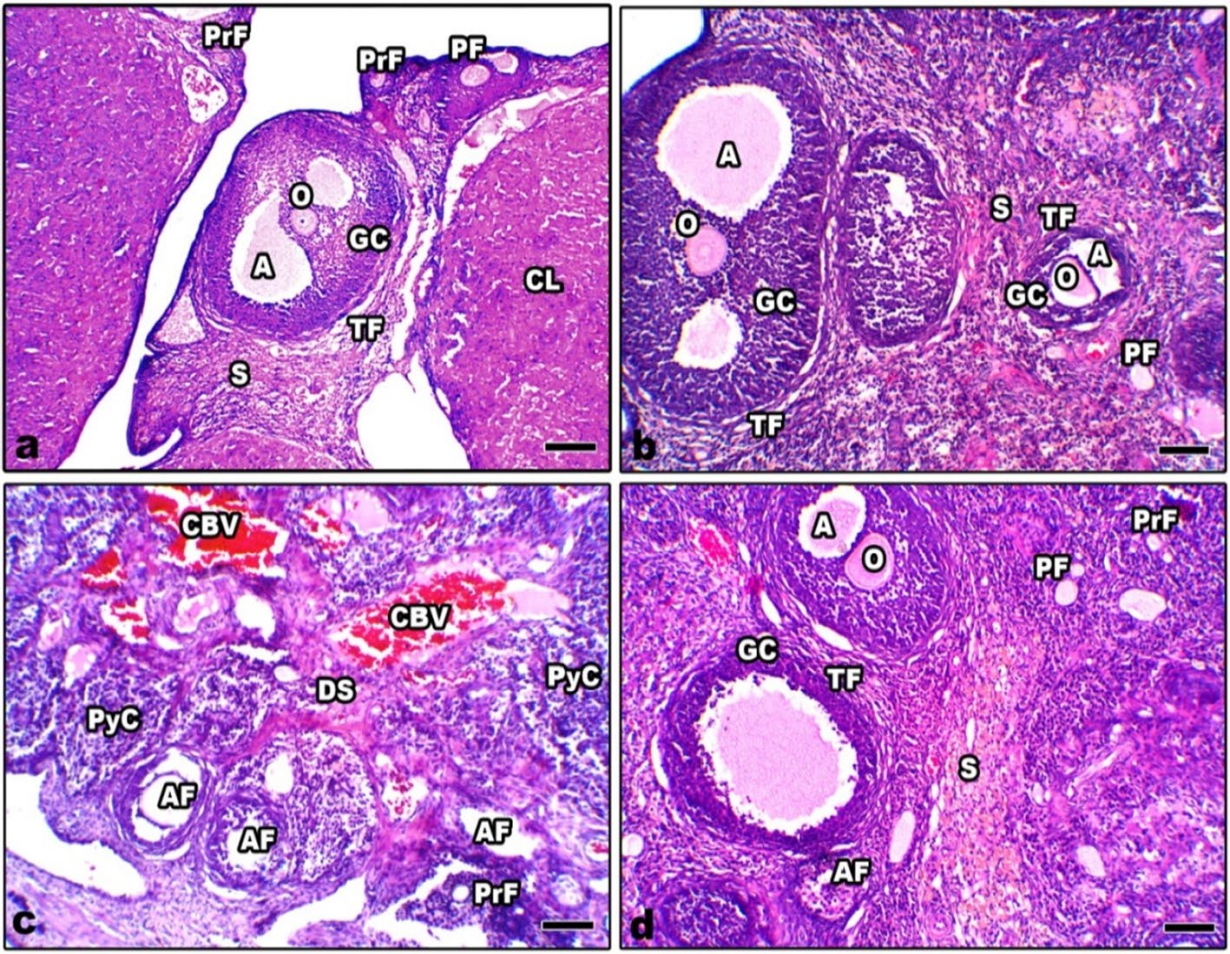
Photomicrograph of histological sections through the ovaries among the different studied groups of mother rats (**a** control, **b** Zn-NP, **c** LPS, and **d** LPS + Zn-NP). The ovarian sections from control and Zn-NP-supplemented pregnant rats appeared to have normal histological architecture. In LPS-treated rats, the ovarian section showed atretic follicles (AF), dispersed pyknotic cells (PyC), and degenerated stroma (DS) with obvious congested blood vessels (CBV). In the LPS group cosupplemented with Zn-NP, the ovarian stroma, as well as the follicles, appear intact, while the little follicles appear atretic (stain H&E, scale bar 25 µm). *A* antrum, *TF* theca folliculi, *GC* granulosa cells, *O* oocyte, *CL* corpus luteum, *S* stroma, *DS* degenerated stroma, *PrF* primordial follicles, *PF* primary follicles, *AF* atretic follicles, *PyC* pyknotic cells, and *CBV* congested blood vessels

The histological sections of the ovaries from control and Zn-NP-supplemented mother rats appeared to have a normal histological pattern, including inner stroma, peripheral follicles, and corpus lutea in different stages (Fig. 2a, b). The ovarian sections from LPS-treated female pregnant rats revealed deleterious histopathological signs, including degenerated ovarian stroma with pronounced dilated and congested blood vessels. Most primary and mature follicles appeared atretic with lysed oocytes and pyknotic cells (Fig. 2c). On the other hand, the ovarian sections from LPS-treated rats cosupplemented with Zn-NP displayed obvious recovery of their histological architecture; the stroma and most of the ovarian follicles appeared well organized while the little follicles were still atretic (Fig. 2d).

**Fig. 3:**
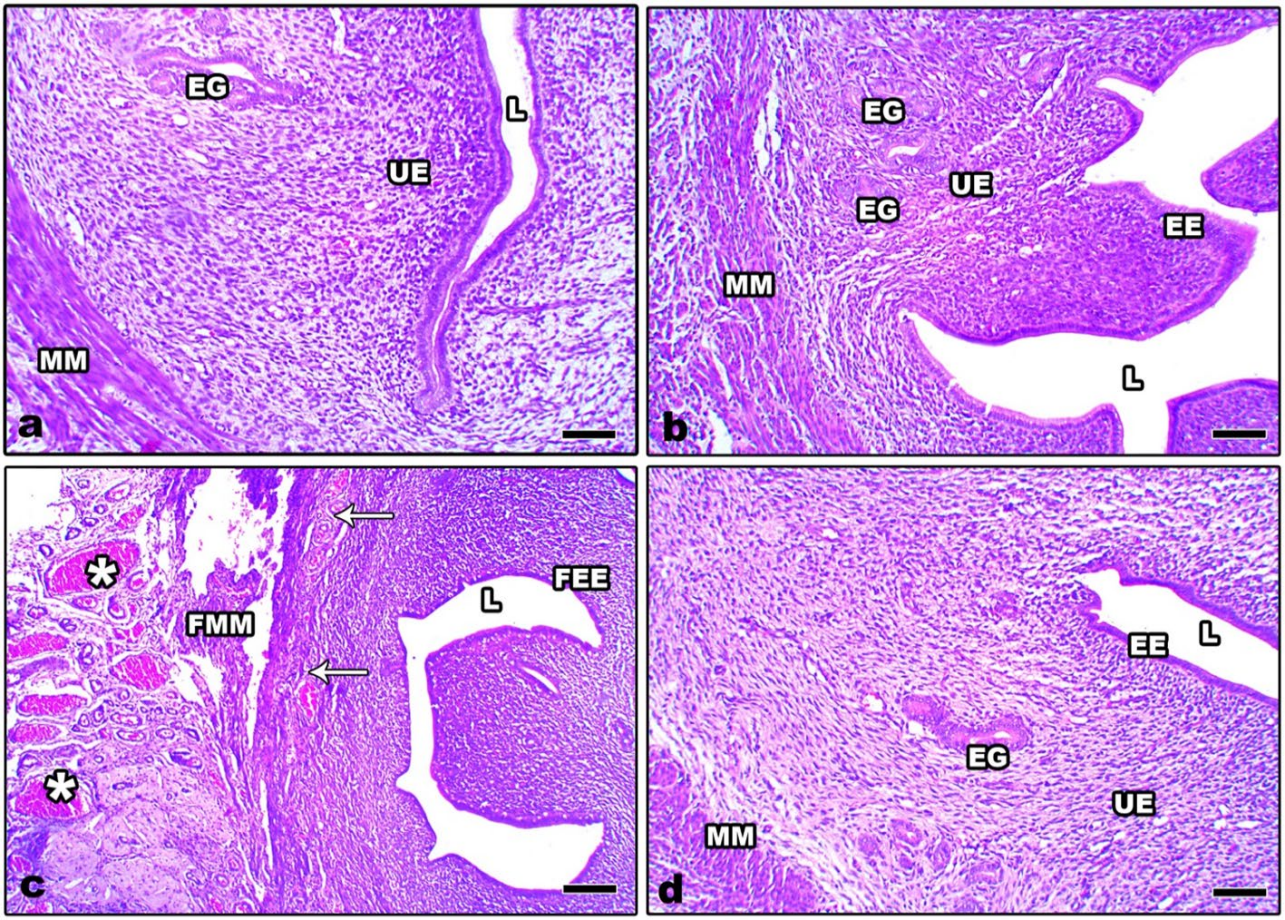
Photomicrograph of histological sections through the uterus among the different studied groups of mother rats (**a** control, **b** Zn-NP, **c** LPS, and **d** LPS + Zn-NP). The uterine sections from control and Zn-NP-supplemented rats appear with normal histological architecture. In LPS-treated rats, the uterine section showed congested capillaries (white asterisks) in the myometrium, as well as fragmented myometrial muscle fibers (FMM), fibrotic tissue in endometrium (arrows), and fragmented endometrial epithelium (FEE). In LPS group cosupplemented with Zn-NP, the uterine layers appear well organized (stain: H&E, scale bar: 100 µm). *MM* myometrium, *UE* uterine endometrium, *EE* endometrial epithelium, *EG* endometrial glands, *L* lumen, *FMM* fragmented myometrium, *FEE* fragmented endometrial epithelium

The uterine sections from control and Zn-NP female pregnant rats appeared to have normal histological architecture, including the outer myometrium and inner endometrium (Fig. 3a, b). In LPS-treated female pregnant rats, the uterine myometrium appeared congested with capillaries among the muscle fibers. The endometrium revealed a fibrotic area with prominent loss of glands and a degenerated epithelial lining (Fig. 3c). Supplementation of Zn-NP to LPS-treated female rats successfully restored the histological architecture of the uterus to normal (Fig. 3d).

**Fig. 4:**
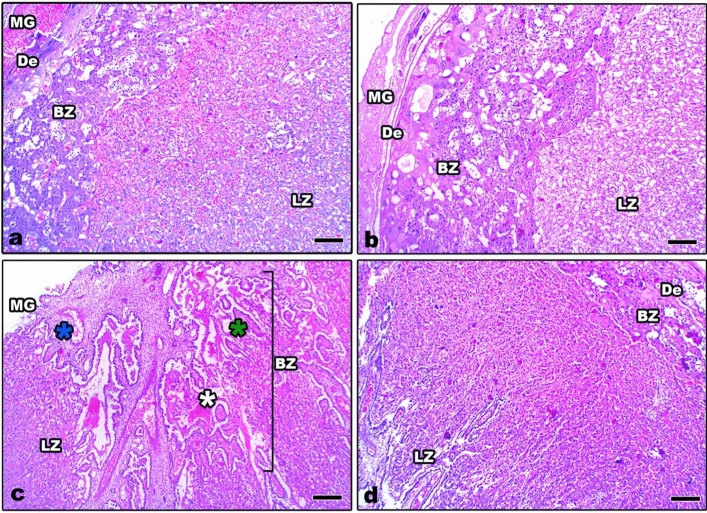
Photomicrograph of histological sections through the placenta among the different studied groups of mother rats at the 16th day of gestation (**a** control, **b** Zn-NP, **c** LPS, and **d** LPS + Zn-NP). The placenta sections from control and Zn-NP-supplemented rats appear to have normal histological architecture. In LPS-treated rats, the placenta layers appear disorganized, with congested capillaries (blue asterisk) and fibrotic tissues (green asterisk). Stain: H & E, scale bar: 25 µm. *MG* metrial glands, *De* decidua, *BZ* basal zone (trophospongium), *LZ* labyrinth zone

In the control and Zn-NP groups, the histological sections of the placenta at the 16th day of gestation appeared well organized into 4 layers; the layer of metrial glands, decidua, basal layer (trophospongium), and labyrinth layer (Fig. 4a, b). In LPS-treated pregnant rats, the layers of placenta appeared disorganized, with prominent congested capillaries and dispersed fibrotic tissues (Fig. 4c). In LPS-treated pregnant rats cosupplemented with Zn-NP, the placenta layers were restored to their normal histological architecture (Fig. 4d).

### Immunohistochemical observations in the ovaries and uterus of mother rats

#### Immunohistochemical localization of Bcl-2

**Fig. 5:**
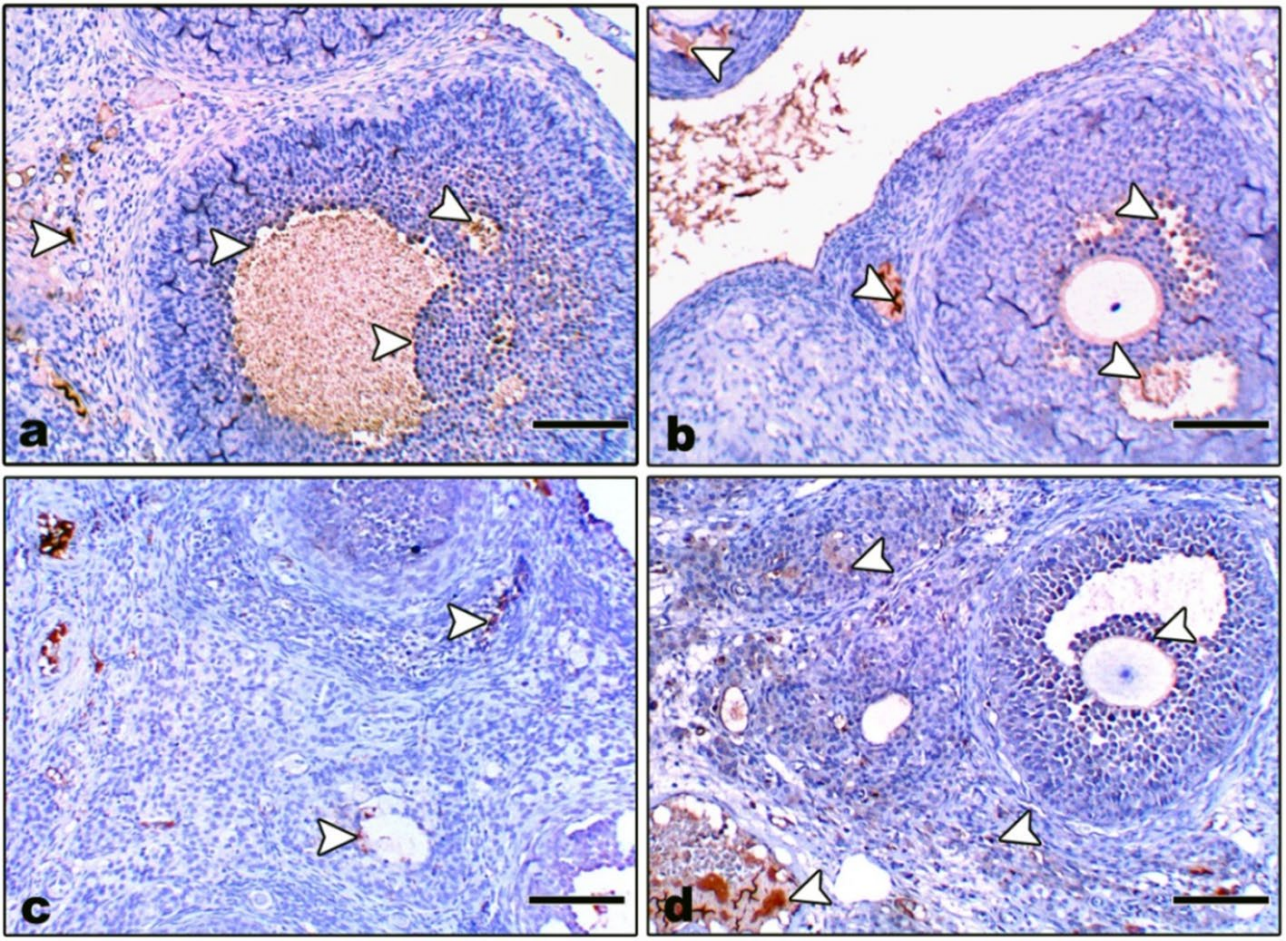
Photomicrograph of paraffin-embedded sections through the ovaries of the different studied groups of female rats stained with Bcl-2 antibody (**a** control, **b** Zn-NP, **c** LPS, and **d** LPS + Zn-NP). Panels (**a** and** b**) display weak to moderate Bcl-2 expression in the follicular cells and little area in the ovarian stroma. In the LPS-treated group (panel **c**), the degree of Bcl-2 immunoreactivity appears negative in the cells of mature follicles and very weak in the ovarian stroma. In LPS-treated rats cosupplemented with Zn-NP (panel **d**), the ovarian stroma shows weak immune expression for Bcl-2, while the follicular cells moderate Bcl-2 expression (Bcl-2 antibody, scale bar: 25 µm). The arrow heads refer to the immunoreactivity of Bcl-2

**Fig. 6:**
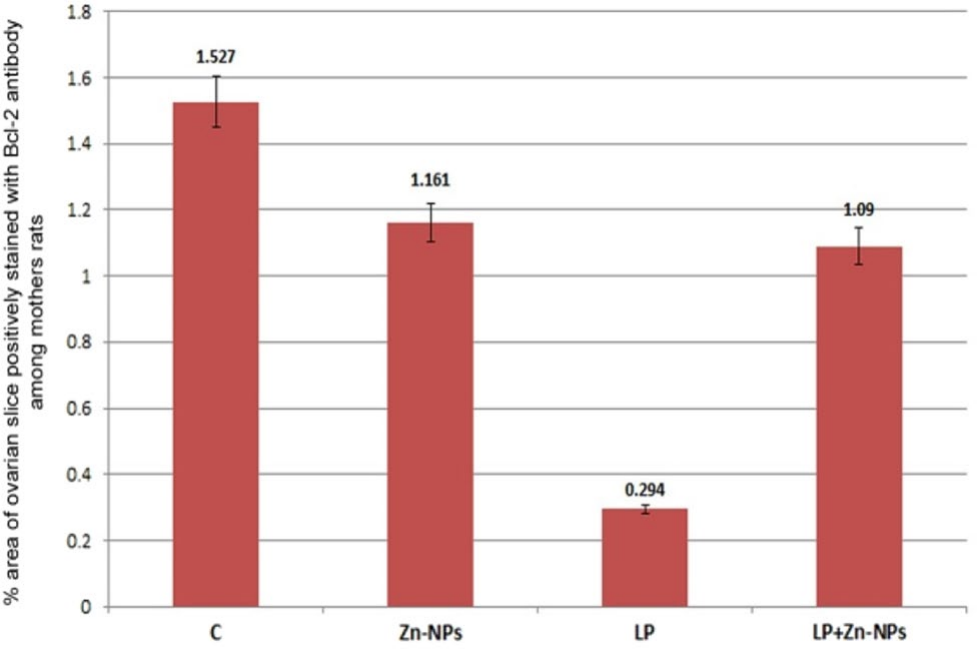
Image analysis for Fig. 5 showing the % of Bcl-2 positively stained area of the ovarian sections among the different studied groups of mother rats

In control and Zn-NP-supplemented female rats, the ovarian sections displayed moderately positive expression for Bcl-2 protein (an antiapoptotic marker). Such expression was more localized in follicular cells and covered little area in the ovarian stroma (Fig. 5a, b). In LPS-treated mother rats, the degree of Bcl-2 immunoreactivity appeared negative in the follicular cells of mature follicles and very weak in the ovarian stroma (Fig. 5c). In LPS-treated rats cosupplemented with Zn-NP, the ovarian stroma displayed weak immune expression for Bcl-2, while the follicular cells appeared moderately stained (Fig. 5d). The percentage area of Bcl-2 positively stained ovarian sections is indicated in Fig. 6.

**Fig. 7:**
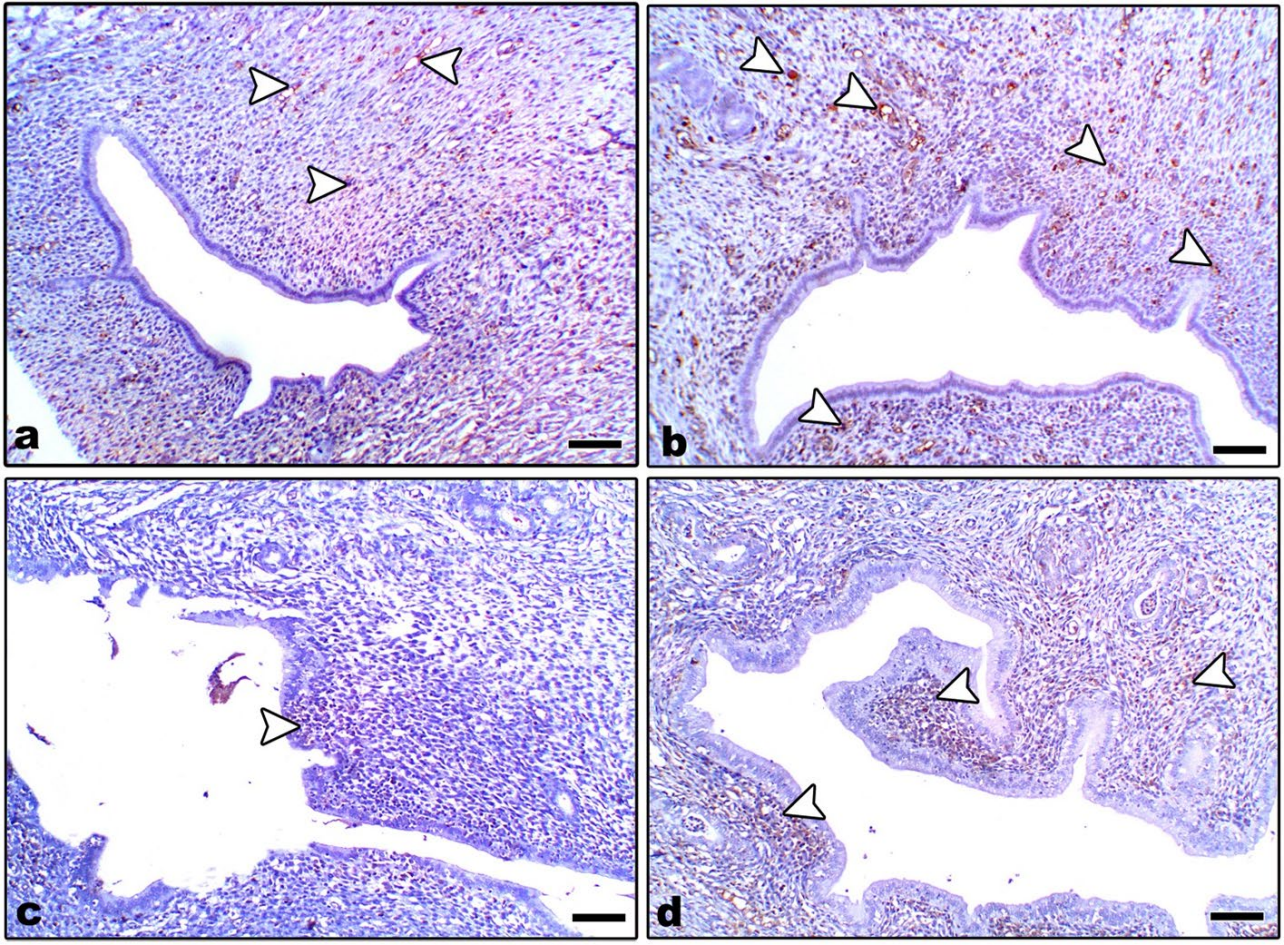
Photomicrograph of paraffin-embedded sections through the uterine endometrium of the different studied groups of female rats stained with Bcl-2 antibody (**a** control, **b** Zn-NP, **c** LPS, and **d** LPS + Zn-NP). The endometrial glands appear moderately stained with Bcl-2 antibody in control and Zn-NP-treated group (panels **a** and** b**), negatively stained in the LPS-treated group (panel **c**), and weakly stained in the LPS + Zn-NP group (panel **d**). Bcl-2 antibody, scale bar: 100 µm. The arrow heads refer to the immunoreactivity of Bcl-2

**Fig. 8:**
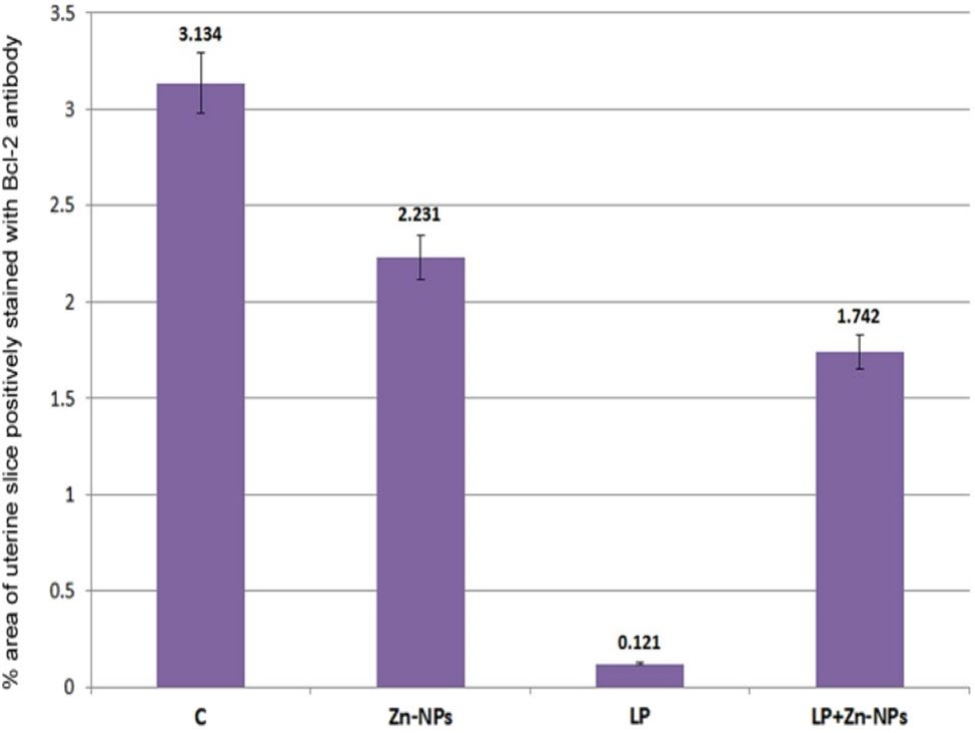
Image analysis for Fig. 7 showing the % of Bcl-2 positively stained area of the uterine sections among the different studied groups of mother rats

Uterine sections from control and Zn-NP-treated mother rats showed moderate Bcl-2 expression, especially in the endometrial glands; however, this expression appeared negative in LPS-treated mother rats. After supplementation of Zn-NP to LPS-treated rats, the degree of immunoreactivity to Bcl-2 was slightly elevated but did not reach that of control (Fig. 7). The percentage area of Bcl-2 positively stained uterine sections is indicated in Fig. 8.

#### Immunohistochemical localization of caspase-3

**Fig. 9:**
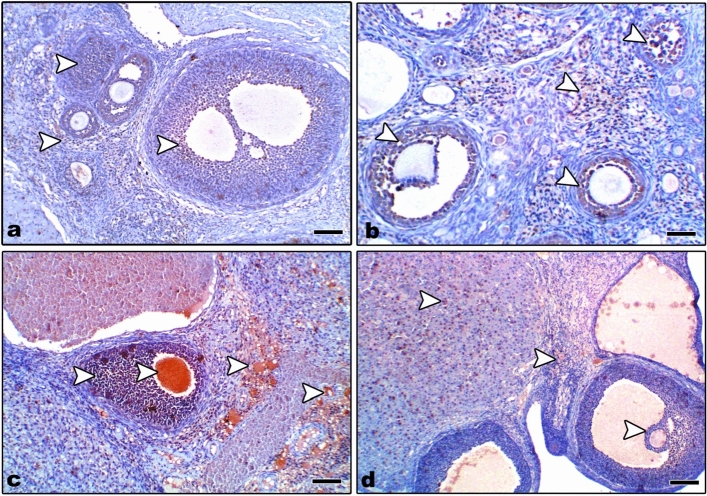
Photomicrograph of paraffin-embedded sections through the ovaries of the different studied groups of female rats stained with caspase-3 antibody (**a** control, **b** Zn-NP, **c** LPS, and **d** LPS + Zn-NP). The follicular granulosa cells and ovarian stroma appear weakly stained with caspase-3 antibody in control and Zn-NP (panels **a** and** b**), strongly stained in the LPS-treated group (panel **c**), and weakly stained in the stroma but negatively stained granulosa cells in the LPS + Zn-NP group (panel **d**). Caspase-3 antibody, scale bar: 25 µm. The arrow heads refer to the immunoreactivity of caspase-3

**Fig. 10:**
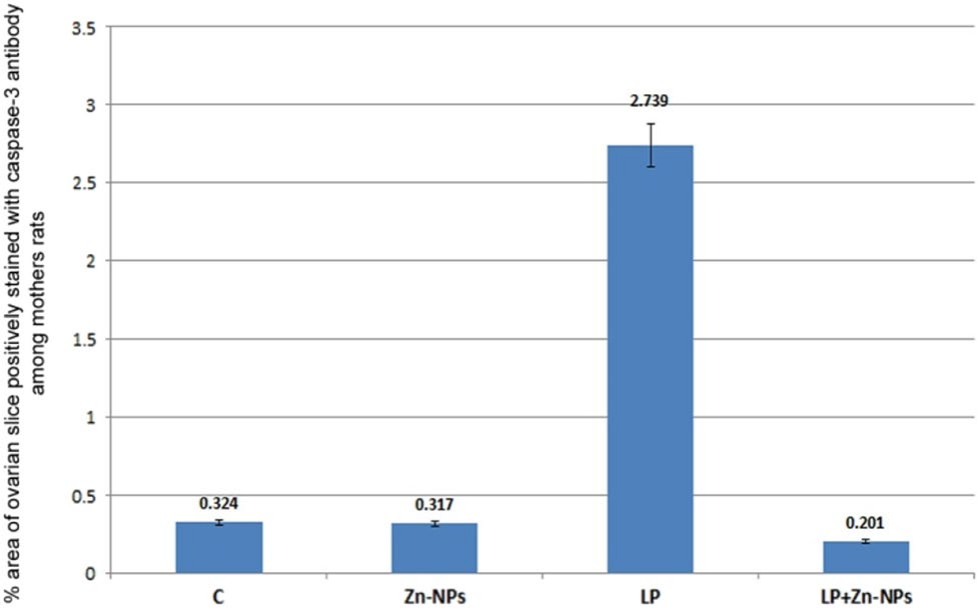
Image analysis for Fig. 9 showing the % of caspase-3 positively stained area of the ovarian sections among the different studied groups of mother rats

In control and Zn-NP-supplemented female rats, the ovarian sections displayed weak positive expression for caspase-3 protein (an apoptotic marker); this expression was more localized in follicular granulosa cells and a small area in the ovarian stroma (Fig. 9a, b). However, in LPS-treated mother rats, the ovarian section appeared to have a stronger expression of caspase-3 when compared with the control (Fig. 9c). In LPS-treated rats cosupplemented with Zn-NP, the ovarian stroma displayed weak immune expression for caspase-3, while the follicular cells appeared negatively stained (Fig. 9d). The percentage area of caspase-3 positively stained ovarian sections is indicated in Fig. 10.

**Fig. 11:**
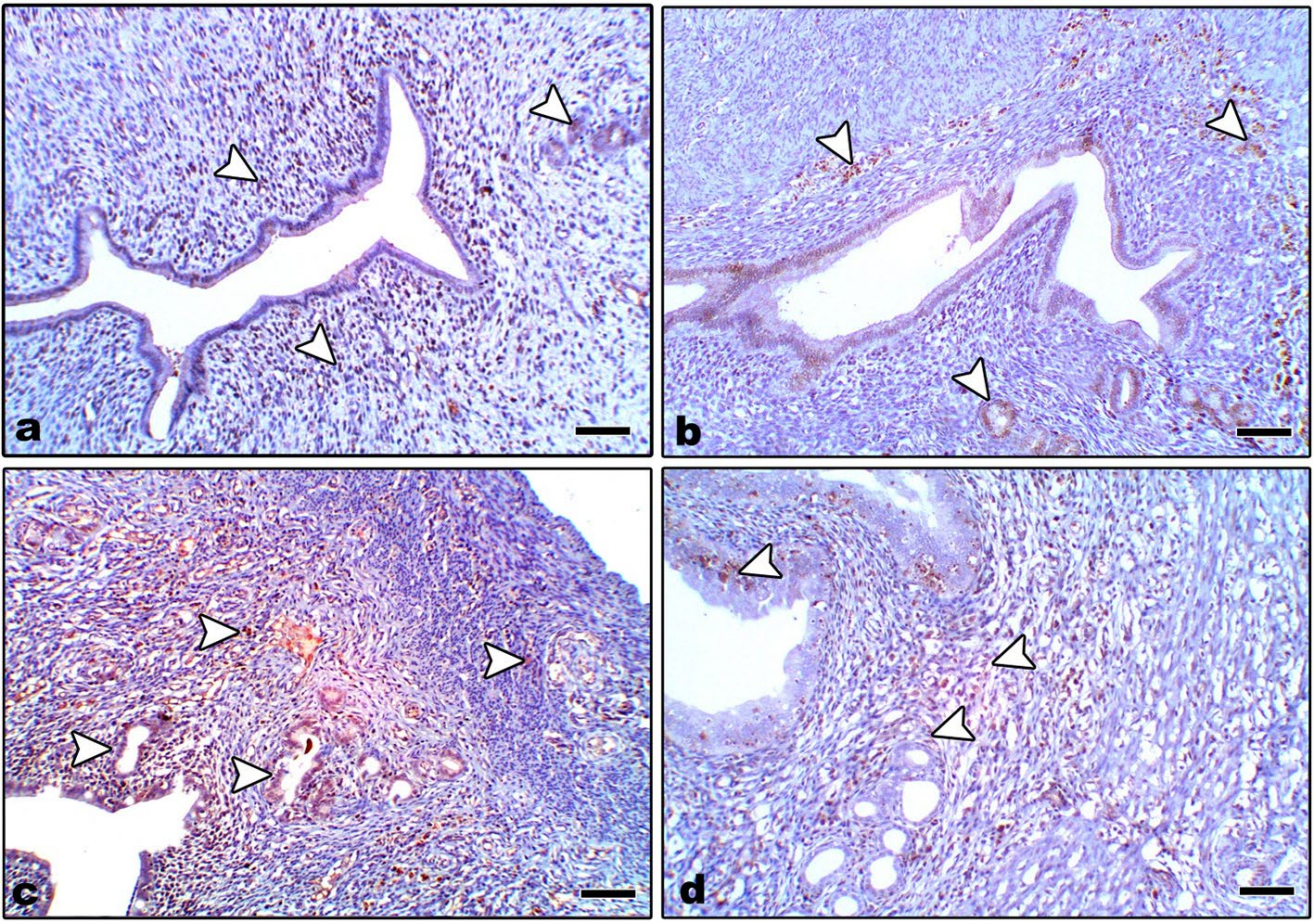
Photomicrograph of paraffin-embedded sections through the uterine endometrium of the different studied groups of female rats stained with caspase-3 antibody (**a** control, **b** Zn-NP, **c** LPS, and **d** LPS + Zn-NP). The endometrial glands appear weakly stained with caspase-3 antibody in control and Zn-NP (panels **a** and **b**), strongly stained in the LPS-treated group (panel **c**), and moderately stained in the LPS + Zn-NpP group (panel **d**). Caspase-3 antibody, scale bar: 100 µm. The arrow heads refer to the immunoreactivity of caspase-3

**Fig. 12:**
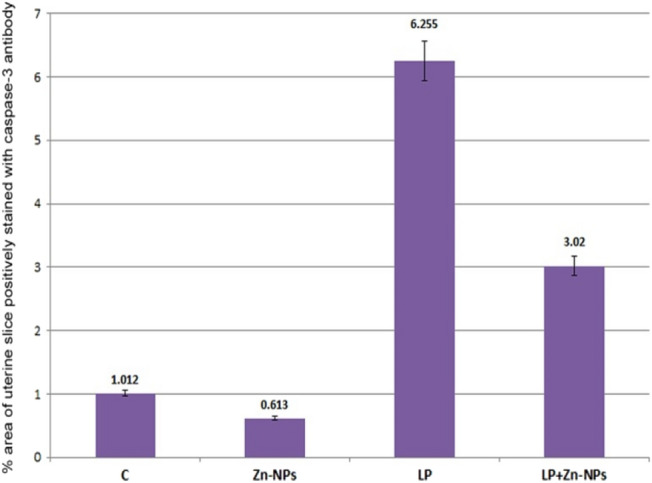
Image analysis for Fig. 11 showing the % of caspase-3 positively stained area of the uterine sections among the different studied groups of mother rats

Uterine sections from control and Zn-NP-treated mother rats showed weak caspase-3 expression, especially in the endometrial glands; however, this expression appeared strongly in LPS-treated mother rats. After supplementation of Zn-NP to LPS-treated rats, the degree of immunoreactivity to caspase-3 appeared moderately expressed but did not reach that of control (Fig. 11). The percentage area of caspase-3 positively stained ovarian sections is indicated in Fig. 12.

### Changes in the levels of serum SOD, CAT, MDA and FSH among mother rats

**Fig. 13:**
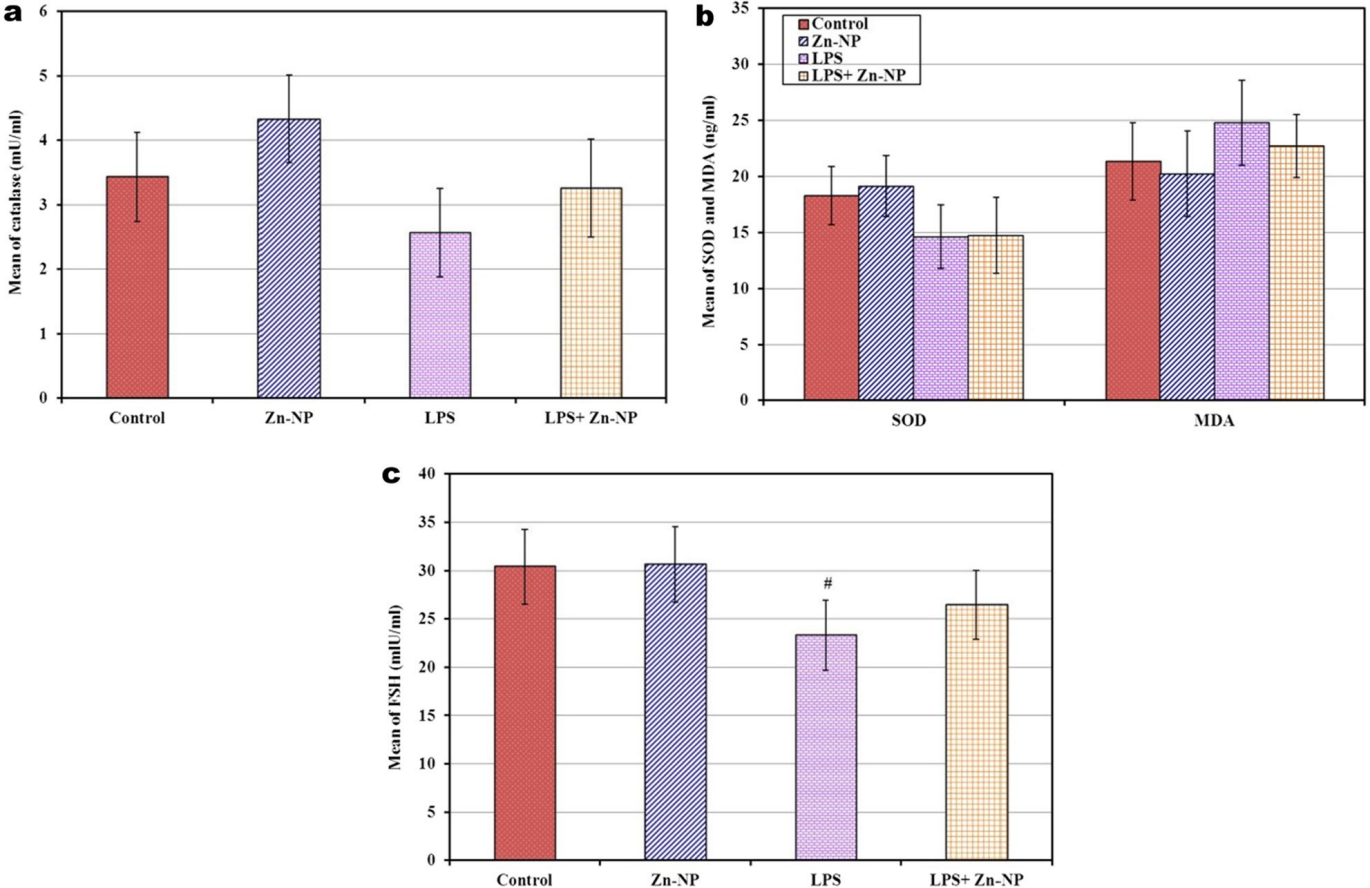
Shows the levels of serum CAT (**a**), SOD and MDA (**b**), and FSH (**c**) among the different studied groups of mother rats

In the control and Zn-NP groups of mother rats, the levels of serum CAT, SOD, MDA, and FSH appeared to be in the normal standard range. In LPS-treated female rats, the levels of serum SOD, CAT, and FSH appeared significantly lower, while the level of MDA appeared significantly higher (*P* < 0.001) than the control. In LPS-treated mother rats cosupplemented with Zn-NP, the levels of CAT, MDA, and FSH were markedly ameliorated and became more or less near the normal values of the control; however, the level of serum SOD did not ameliorate and is still significantly lower (*P* < 0.001) than control (Fig. 13).

### Histopathological changes in the ovaries of 21 day old offspring

**Fig. 14:**
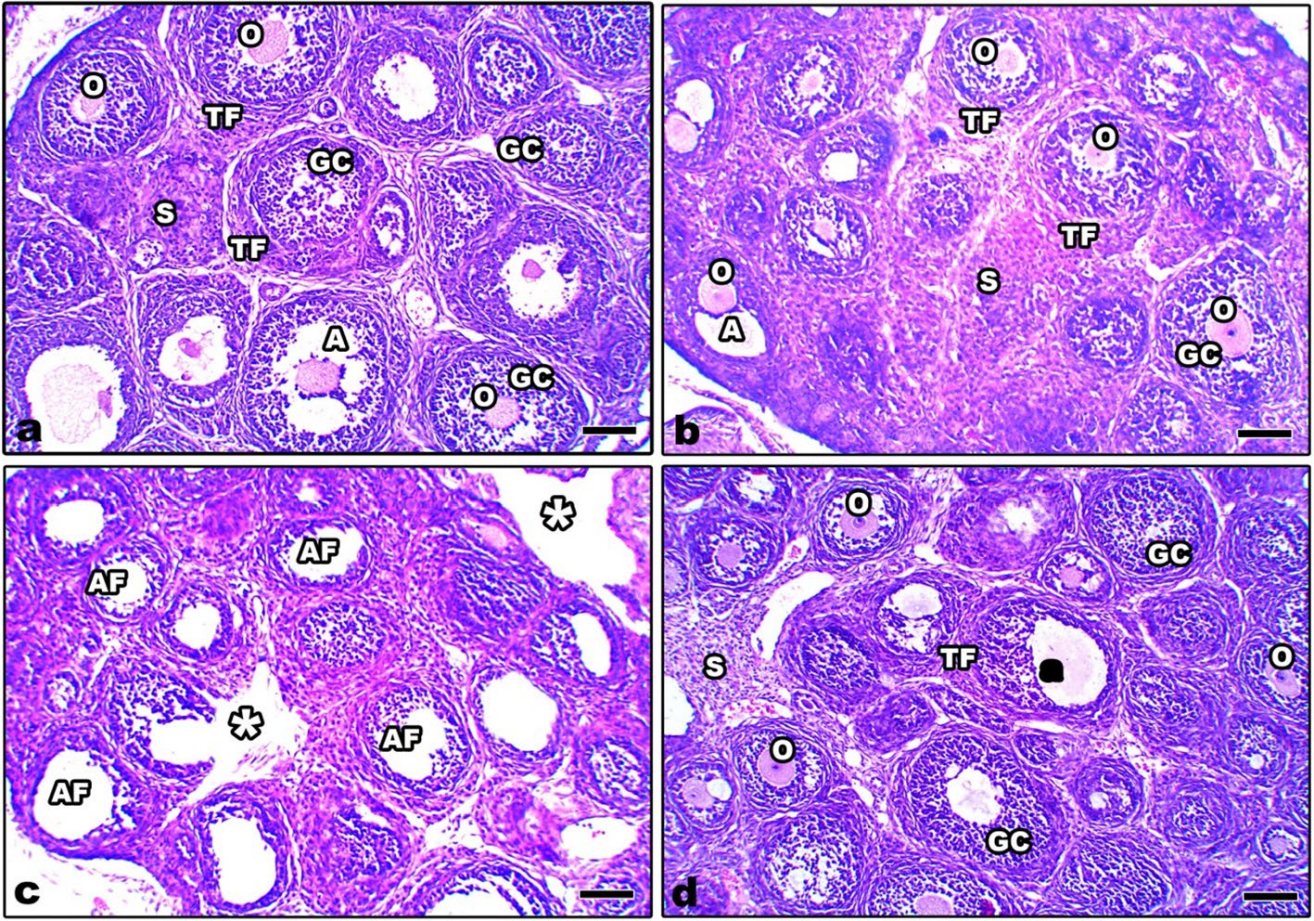
Photomicrograph of histological sections through the ovaries among the different studied groups of 21-day-old offspring (**a** control, **b** Zn-NP, **c** LPS, and **d** LPS + Zn-NP). In the control and Zn-NP groups, the ovarian sections appear to have a well-organized pattern of ovarian follicles embedded in the ovarian stroma. In the LPS group, the ovarian follicles appear to be few, and most of them are atretic. In LPS + Zn-NP, the ovarian follicles show remarkable amelioration in their architecture. Stain: H&E, scale bar: 25 µm. *A* antrum, *GC* granulosa cells, *O* oocyte, *TF* theca folliculi, *S* stroma, *AF* atretic follicles, * ruptured follicles

The ovarian sections from control and Zn-NP maternally supplemented 21-day-old offspring showed a high density of well-organized and well-preserved ovarian follicles (Fig. 14a, b). The ovarian follicles appeared rounded with a centrally located oocyte and surrounded by two compact layers: theca folliculi and inner granulosa cells. In LPS maternally treated offspring, the ovarian follicles appeared few in number, they lost their oocytes, and the follicular layers appeared atretic (Fig. 14c). In Zn-NP maternally treated offspring, the deleterious histological signs of ovarian follicles caused by LPS were restored almost to normal follicles, as compared to the control (Fig. 14d).

### Immunohistochemical observations in the ovaries of 21 day old offspring

#### Immunohistochemical localization of Bcl-2

**Fig. 15:**
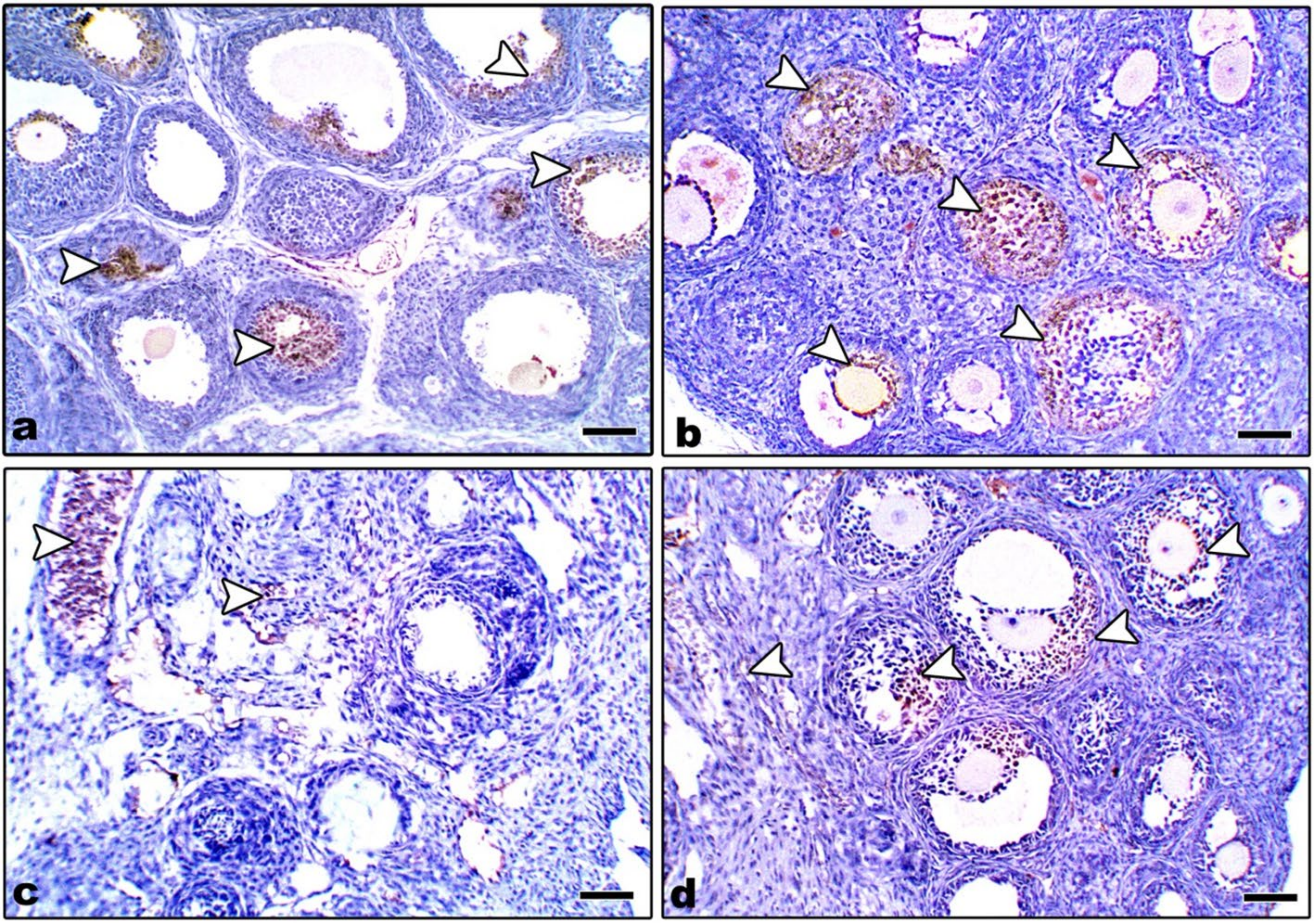
Photomicrograph of paraffin-embedded sections through the ovaries of the different studied groups of 21-day-old offspring stained with Bcl-2 antibody (**a** control, **b** Zn-NP, **c** LPS, and **d** LPS + Zn-NP). In control and Zn-NP (panels **a** and** b**), the ovarian sections display moderate Bcl-2 expression in the follicular cells. In the LPS-treated group (panel **c**), the degree of Bcl-2 immunoreactivity appears very weak in the cells of some follicles. In LPS-treated rats cosupplemented with Zn-NP (panel **d**), the follicular cells show moderate Bcl-2 expression. In all groups, the ovarian stroma appeared negatively stained for Bcl-2 protein (Bcl-2 antibody, scale bar: 25 µm).The arrow heads refer to the immunoreactivity of Bcl-2

**Fig. 16:**
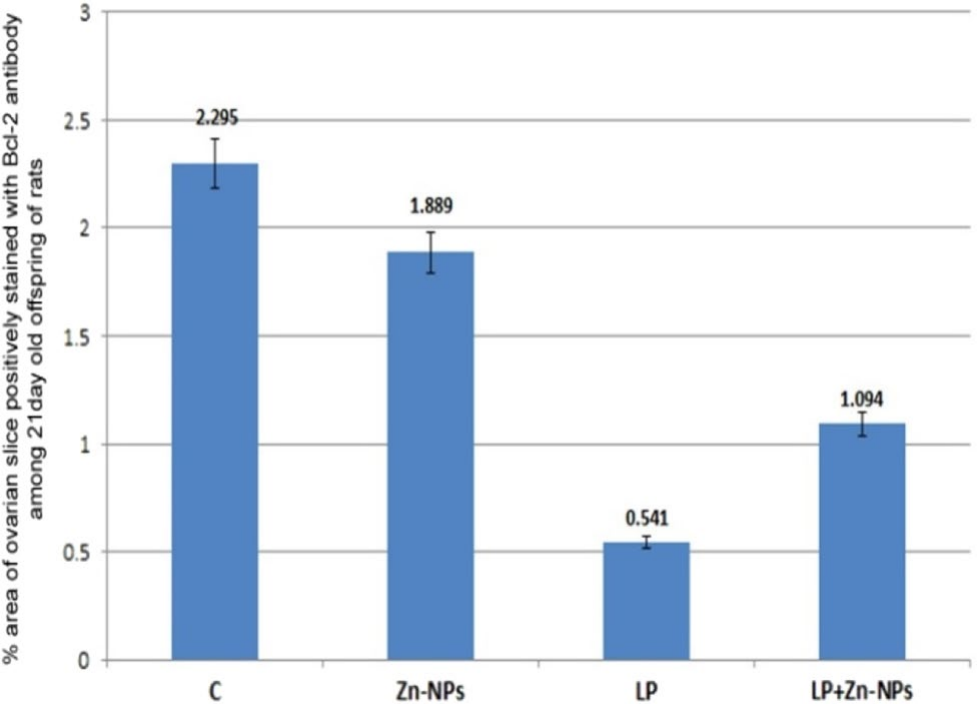
Image analysis for Fig. 15 showing the % of Bcl-2positivelly stained area of the ovarian sections among the different studied groups of 21 day old offspring

In control and Zn-NP maternally supplemented 21-day-old offspring, a moderately positive Bcl-2 expression was recorded in the granulosa cells of ovarian follicles, while the stroma appeared negatively stained. However, the ovarian section from LPS maternally treated offspring revealed very weak expression for Bcl-2 in some follicles. In 21-day-old offspring maternally treated with LPS and cosupplemented with Zn-NP, the follicular granulosa cells showed weak Bcl-2 expression and the stroma appeared negatively stained (Fig. 15). The percentage of Bcl-2-positively stained areas of the ovarian sections was indicated in Fig. 16.

#### Immunohistochemical localization of caspase-3

**Fig. 17:**
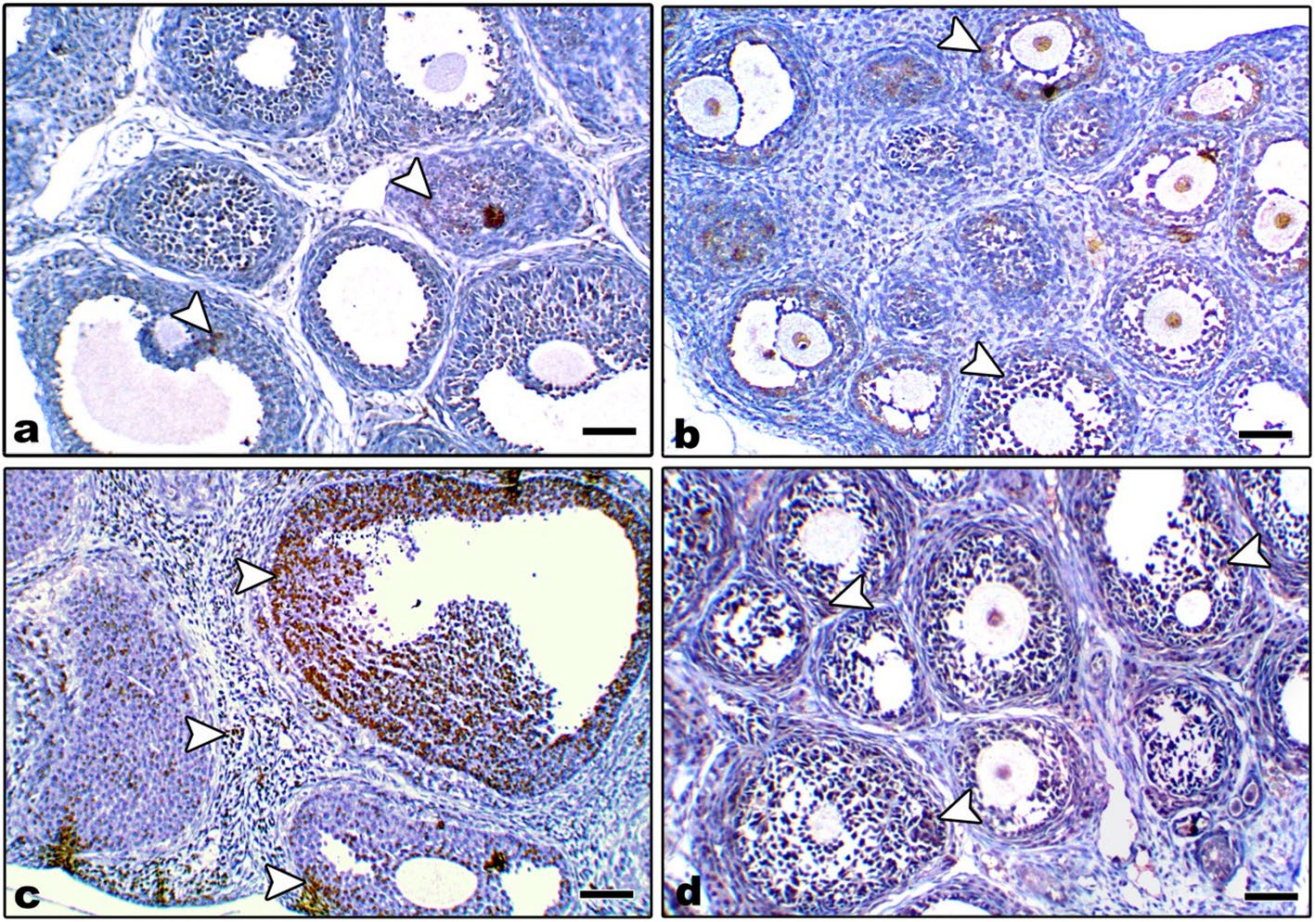
Photomicrograph of paraffin-embedded sections through the ovaries of the different studied groups of 21-day-old offspring stained with caspase-3 antibody (**a** control, **b** Zn-NP, **c** LPS, and **d** LPS + Zn-NP). In control and Zn-NP groups (panels **a** and** b**), the immunoreactivity for caspase-3 appears very weak in the follicular cells and negative in the ovarian stroma. In the LPS-treated group (panel **c**), the degree of caspase-3 immunoreactivity appears strongly expressed in both follicular cells and the ovarian stroma. In LPS-treated rats cosupplemented with Zn-NP (panel **d**), the caspase-3 activity appears very weak in the follicular cells and negative in the ovarian stroma. Caspase-3 antibody, scale bar: 25 µm. The arrow heads refer to the immunoreactivity of caspase-3

**Fig. 18:**
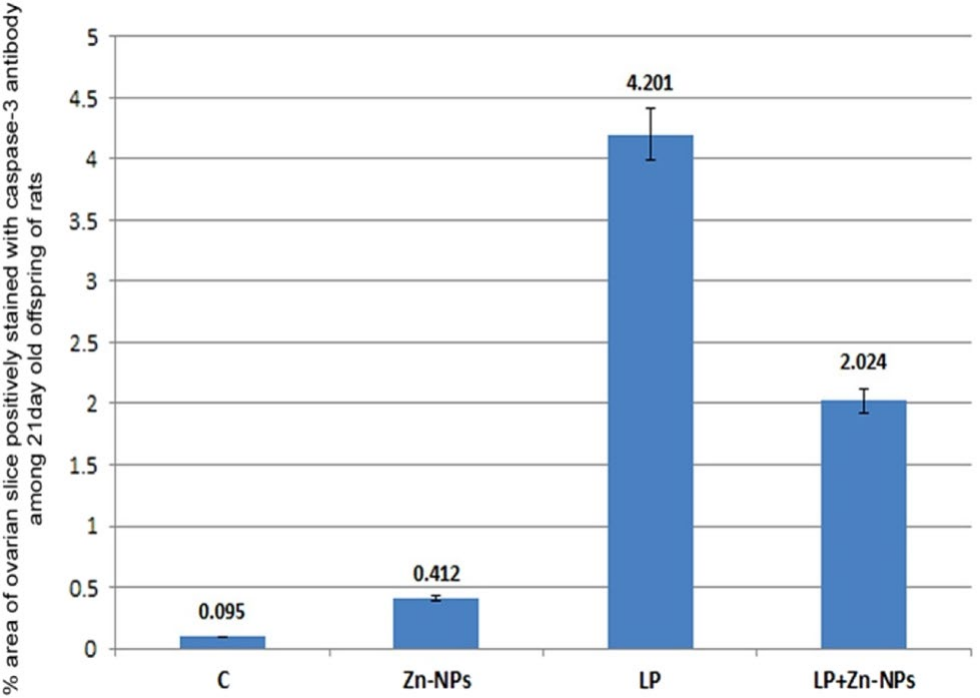
Image analysis for Fig. 17 showing the % of caspase-3 positively stained area of the ovarian sections among the different studied groups of 21-day-old offspring

In control and Zn-NP maternally supplemented 21-day-old offspring, a very weak positive caspase-3 expression was recorded in the granulosa cells of ovarian follicles, while the ovarian stroma appeared negatively stained. However, a strong positive reaction was recorded in the granulosa cells, as well as in the ovarian stroma of LPS-treated offspring. In 21-day-old offspring maternally treated with LPS and cosupplemented with Zn-NP, the follicular granulosa cells showed very weak caspase-3 expression, and the stroma appeared negatively stained (Fig. 17). The percentage of caspase-3 positively stained areas of the ovarian sections was indicated in Fig. 18.

### Changes in the levels of SOD, CAT, and MDA, in the ovarian tissues among 21-day-old offspring

**Fig. 19:**
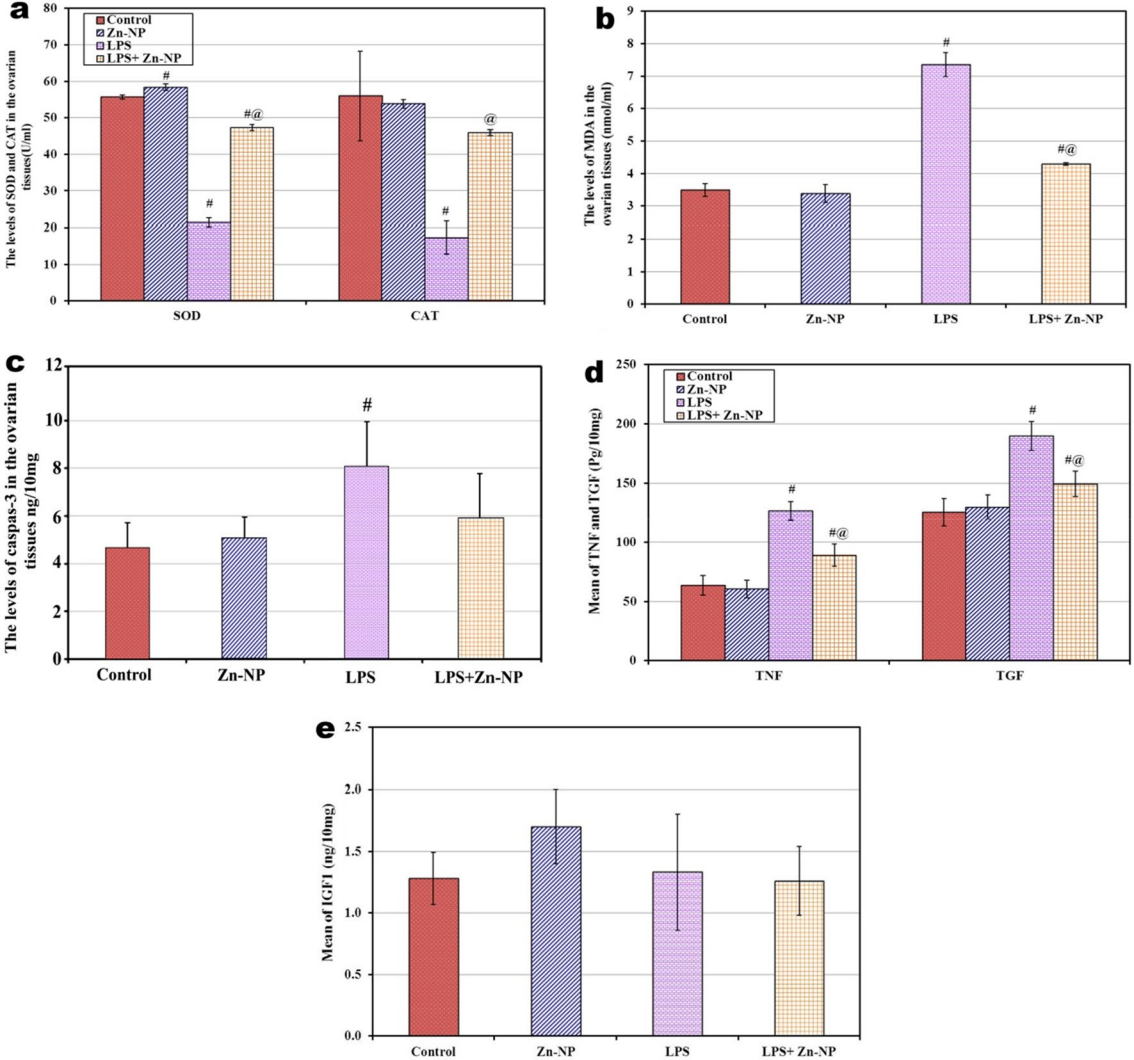
Changes in the levels of serum SOD, CAT (panel** a**), MDA (panel** b**), caspase-3 (panel **c**), TNF-α, TGF-β1 (panel **d),** and IGF-1 (panel **e**) among the different studied groups of 21-day-old offspring

In Zn-NP-supplemented female offspring, the ovarian levels of CAT and MDA appeared to show a non-significant change compared to control; however, the level of SOD appeared significantly higher (*P* < 0.001) than the control. On the other hand, in LPS maternally treated offspring, the levels of ovarian SOD and CAT were significantly lowered (*P* < 0.001) while the level of MDA was significantly higher (*P* < 0.001) when compared with the control. In offspring maternally supplemented with LPS and Zn-NP, the levels of SOD and CAT were significantly elevated (*P* < 0.001), while the level of MDA was significantly lower than when compared with the LPS group alone but still showed a significant change with control (Fig. 19a, b).

### Changes in the levels of caspase-3, IGF-1, TNF-α, and TGF-β1 in the ovarian tissues of 21-day-old offspring

In offspring maternally treated with Zn-NP alone, the level of caspase-3 in the ovarian tissues appeared to be similar to that of control; however, in offspring maternally treated with LPS, the level of caspase-3 appeared significantly higher (*P* < 0.001) than the control. On the other hand, in offspring maternally treated with LPS and Zn-NP, the level of caspase-3 in the ovarian tissue showed no significant change with control (Fig. 19c).

The levels of IGF-1 in the ovarian tissue appeared to show no significant change among all studied groups, however, the levels of TNF-α and TGF-β1 appeared significantly higher in LPS maternally treated offspring when compared with the control. On the other hand, the levels of TNF-α and TGF-β1 were significantly decreased (*P* < 0.001) in the LPS + Zn-NP group of offspring when compared with the LPS group of offspring, but still significantly higher (*P* < 0.001) than the control (Fig. 19d, e).

## Discussion

Microbial infections during gestation are one of the main causes of preterm labor and adverse fetal outcomes, which persist as major and unresolved challenges. Systemic maternal LPS exposure has been previously shown to be associated with adverse fetal developmental outcomes in both humans and animals (O’Sullivan et al. 1988). LPS was reported to induce inflammation in human gestational tissues, resulting in an increase in the release of proinflammatory cytokines (Fortunato et al. 1996) and phospholipid metabolites (Mitchell et al. 1993). Zinc (Zn) is an essential trace element required for the maintenance of germ cells in gonads. Zn deficiency in the mother can be inherited by the infant (Nenkova et al. 2017). Zinc nanoparticles (Zn-NPs) are the new forms of the material with prominent biological properties and low toxicity that appear to have excessive potential to overcome some of the barriers regarding efficient targeting of cells and molecules in many diseases (Suri et al. 2007). Pelgrift and Friedman declared that Zn-NPs could overcome microbial resistance (Pelgrift and Friedman 2013). Lallo da Silva et al. added that the antimicrobial activities of Zn-NPs increased with decreasing their particle size or increasing their surface area (Lallo da Silva et al. 2019). Accordingly, the current study was mainly designed to evaluate the potential ameliorative role of Zn-NP against LPS-induced toxicity in pregnant rats during perinatal life.

The obtained results revealed a significant decrease in the body weight of the LPS-exposed pregnant rats at E16 of gestation and at 21 days postnatal when compared with the control; however, after supplementation with Zn-NP, the body weights were obviously restored near normal. The obtained results go parallel with the finding of Wang et al. who found that exposure to LPS during mid-gestation is accompanied by a significant decrease in the body weight of pregnant mice and their fetuses (Wang et al. 2014). The decreased body weight in LPS-exposed pregnant rats may be attributed to low food intake, whereas the LPS may participate in loss of appetite (Desai et al. 2007). Further conformational explanation to our obtained results: the placental weight appeared significantly lowered in LPS-exposed pregnant rats when compared with the control. The decreased placental weight of LPS-exposed pregnant rats reflected the decreased blood supply to the fetuses, resulting in fetal growth retardation. Restoration of body weight to near normal value in LPS-exposed pregnant rats after supplementation with Zn-NP is in accordance with Pei et al., who reported that low doses of Zn-NP play an essential role in growth performance through enhancement of food intake and regular assimilation by intestinal enzymes (Pei et al. 2019).

Oxidative stress is a condition in which an imbalance occurs between the production of reactive oxygen species (ROS) and their elimination by antioxidant protective mechanisms. It is widely accepted that inflammation is associated with oxidative stress and that elevated levels of intracellular ROS are the most potent mediators of inflammation (Zhang et al. 2019). Recently, it has been reported that exposure to LPS leads to the activation of macrophages, which results in excessive production of ROS (Hseu et al. 2022). ROS overproduction also plays a key role in inflammation (Tschopp and Schroder 2010). In the current work, LPS exposure during gestation is implicated in the reduction of serum SOD and CAT levels, while the level of serum lipid peroxidation (MDA) appeared significantly elevated when compared with the control. In LPS-treated rats cosupplemented with Zn-NP, the levels of CAT and MDA were markedly restored to near the normal value of the control; however, the level of serum SOD did not ameliorate and is still significantly lower than control. The depletion of endogenous antioxidant enzymes has been shown in the brains of LPS-treated pregnant rats (Sebai et al. 2009). Other studies have reported that LPS can induce lipid peroxidation in vitro (Stuss et al. 2010) and in vivo (Requintina and Oxenkrug 2003) studies.

The obtained results showed severe histopathological signs in the ovaries, uterus, and placenta of LPS-exposed pregnant rats. These signs included atretic ovarian follicles, dispersed pyknotic cells, and damaged stroma with obvious congested blood vessels. The uterine histopathological signs are represented by congested capillaries in the myometrium, fragmented myometrial muscle fibers, fibrotic tissue in the endometrium, and fragmented endometrial epithelium. Furthermore, the placenta layers appeared disorganized, with congested capillaries and fibrotic tissues. Similar observations were recorded in the ovaries of mice (Shokrizadeh et al. 2019; Lv et al. 2021), the ovaries and uteruses of cows (Magata 2020), the uteruses of mice (Jaiswal et al. 2009; Wolfson et al. 2015), and the placenta of mice (Fricke et al. 2018). Previous research reported that prenatal LPS exposure is implicated in the production of toxic free radicals (^.^OH) (Cambonie et al. 2004) and an increased level of superoxide anion (O^2−^) (Bautista et al. 1990). Free radicals react with important biological substrates, including DNA, proteins, and lipids, which interrupt cell function and may lead to cell injury. Other studies revealed that ROS cause cellular damage, including lipid peroxidation, thus interrupting the fluidity of the cell membrane (Al-Amin et al. 2016). Additionally, it has been reported that LPS exposure during gestation is implicated in the release of excessive cytokines that alter the structure and function of placental cells. The atretic follicles that appeared in this study may be attributed to the fact that LPS-induced mitochondrial damage in the oocyte, as previously reported by Stojkovic et al. (Stojkovic et al. 2001). Magata added that LPS exposure during gestation results in reduced mitochondrial membrane potential in mature oocytes and consequently leads to follicular damage (Magata 2020). The histopathological alterations in the uterus of LPS-treated rats were recently explained by Lu et al., who reported that LPS exposure during gestation can induce glandular and epithelial damage of the uterine endometrium via inactivation of E-cadherin expression (Lu et al. 2021). E-cadherin is the most important member of the cadherin family involved in cell adhesion and plays an extremely important role in the adhesion between cells and in maintaining the morphological structure of tissues (Furuse and Tsukita 2006). Recently, it was found that LPS can induce severe histopathological signs in the liver, kidneys, and ovaries of female rats via the induction of oxidative stress and DNA damage (Aboelmaaty et al. 2022).

Administration of Zn-NPs during gestation and lactation to pregnant rats successfully alleviated the ovarian, uterine, and placental histological changes and oxidative stress induced by LPS. Studies have shown that Zn can pass through the placenta via the microvilli in the syncytiotrophoblast (Donangelo and King 2012). Till now, no previous work has focused on the use of Zn-NPs in enhancing reproductive organs in pregnant rats, but other related studies have declared that zinc supplementation during gestation and lactation periods plays an essential role in improving the fetus’s antioxidant levels (Jafari et al. 2017). Previous reports revealed that Zn is a powerful antioxidant agent (Ahmad et al. 2013), which can inhibit oxidative stress by scavenging free radicals and reducing lipid peroxidation (Bray and Bettger 1990). MacDonald added that zinc plays a role in cell proliferation, has an ameliorative effect on the cell membrane, and is responsible for secondary cell signaling (MacDonald 2000). Accordingly, the ameliorative effect of Zn-NPs against the deleterious histological changes induced by LPS in the reproductive organs of female rats may be attributed to the direct antioxidant properties of Zn (Ozcelik et al. 2012) and to the production of endogenous antioxidants, which are produced by the cell itself (Ahmad et al. 2013) and are able to prevent cell damage from free radicals.

The results of the present work revealed that LPS can induce apoptosis in the follicular cells of the ovaries and endometrial tissues of female rats via strong expression of caspase-3 (an apoptotic marker) and weak expression of Bcl-2 (an antiapoptotic marker). Similar observations were recorded in the ovarian tissues of LPS-exposed bovine (Luttgenau et al. 2016) uterine tissue (Chanrot et al. 2017), and placental cells (Ejima et al. 2000). Previous reports revealed that LPS administration can induce apoptosis in the trout ovary through activation of ZIP-kinase (a positive mediator of apoptosis) (Kawai et al. 1998; MacKenzie et al. 2006) and decrease the expression of several anti-apoptotic genes (Liang et al. 1998). Another related study evaluated that LPS can induce cell death that is mediated by the accumulation of reactive oxygen species and activation of p38 in the rat cortex and hippocampus (Nolan et al. 2003).

Zinc nanoparticles successfully alleviated the degree of apoptosis in the ovarian and uterine cells via inactivation of caspase-3 expression and activation of Bcl-2 activity. Similar studies revealed the antiapoptotic and antioxidant effects of zinc against cadmium-induced apoptosis in the reproductive organs of males and females (Chimienti et al. 2003; Ebisch et al. 2007). Other studies found that zinc supplementation can inhibit apoptosis and increase antioxidants in the testicular tissues induced by diabetes in rats (Afifi et al. 2015; Aziz et al. 2018). As previously reported by Ebisch et al., the antiapoptotic effect of Zn-NPs was mainly attributed to their role in protein synthesis and repair of damaged DNA, as well as the acceleration of germ cell proliferation in the reproductive organs (Ebisch et al. 2007). In the current work, the use of zinc nanoparticles, instead of zinc in its metal form, is more efficient in alleviating deleterious histological and biochemical changes in the reproductive organs of female rats.

The obtained results revealed that LPS maternally exposed offspring have decreased levels of the ovarian antioxidant enzymes SOD and CAT and an increased level of lipid peroxidation (MDA) when compared with control offspring. These findings indicated a correlation between the prenatal administration of LPS and the induction of oxidative stress in the offspring. The obtained results go in parallel with previous reports (Seweryne et al. 2011; Al-Amin et al. 2016), which found that LPS is implicated in the depletion of endogenous antioxidant enzymes like CAT and SOD. A related study revealed that prenatally LPS-exposed rats resulted in a significant reduction in the levels of SOD and CAT in the midbrain of female offspring (Sharma et al. 2017). Furthermore, short- and long-term effects of LPS were found to induce endotoxemia in mouse ovarian tissue (Shokrizadeh et al. 2019). Moreover, LPS injected into pregnant mice affects the behavior of their offspring in adulthood (Chlodzinska et al. 2011). Altogether, these data suggest that prenatal maternal LPS injections generally decrease antioxidants and increase oxidative stress in the ovarian tissues of pups.

Zinc is a major component of metallothioneins. Mmetallothioneins are effective in inhibiting oxidative stress via regulation of the secretion of proinflammatory cytokines (Prasad 2008) and by scavenging free radicals (Greenstock et al. 1987). Furthermore, zinc is a powerful antioxidant factor via free radical scavenging enzymes such as SOD, and a recognized protector of sulfhydryl groups is also thought to impair lipid peroxidation by displacing transition metals such as iron and copper from catalytic sites (Bray and Bettger 1990). In the current study, prenatal and postnatal zinc supplementation was successfully able to restore the activity of antioxidant enzymes suppressed by LPS. The obtained results agree with a related study reported by Sharma et al. who found that zinc supplementation during gestation can alleviate the oxidative stress in LPS maternally treated rats offspring (Sharma et al. 2017).

In the present work, severe histopathological signs were recorded in the ovarian tissues of LPS maternally treated offspring. The ovarian histopathological signs included a small number of follicles; most of them appeared atretic and degenerated. Similar histopathological features were recorded in the ovaries of LPS-treated female mouse offspring (Adetunji et al. 2020). The ovarian histopathological signs that appeared in the offspring maternally treated by LPS may be attributed to the direct oxidative stress on the placenta during gestation. Other reports indicated that the incidence of histopathological signs in the LPS maternally treated offspring is fundamentally attributed to the excess liberation of proinflammatory cytokines such as TNF-α and IL-1β in the amniotic fluid during the gestation period (Huleihel et al. 2004; Hagberg et al. 2005). Further study declared that increased proinflammatory cytokine levels could affect the brain of the developing fetus and may be responsible for the inhibition of the reproductive axis and its normal function in the fetuses (Solati et al. 2012).

Apoptosis is a normal physiological cell death that takes place under controlled mechanisms involving caspase enzymes. Disturbances in apoptotic enzymes lead to stimulation or inhibition of the apoptotic rate. The process of apoptosis is very complex and usually influenced by the caspases and the Bcl-2 family of factors (Srivastava et al. 2015; Takei et al. 2015). Caspase-3 plays an important role in the protease cascade cleavage, which can hydrolyze apoptotic proteins in cells, promoting the apoptosis of such cells. Other pathways, such as the mitochondrial pathway, are also important; when an apoptosis factor stimulates cells, mitochondria can produce apoptosis factors, inducing cell death (Pradeep et al. 2016; Tian et al. 2012). The Bcl-2 family of proteins is prevalent in humans and animals, where its main role is to inhibit apoptosis and promote proliferation. The Bcl-2 gene inhibits apoptosis in three ways (Adefolaju et al. 2015). First, through its action as an antioxidant; second, through its inhibition of the production of endoplasmic reticulum calcium ions; and finally, through its interaction with other apoptosis factors such as Bax. Activated Bcl-2 can promote the migration and proliferation of endothelial cells and inhibit apoptosis (Aliparasti et al. 2015; Wright et al. 2015). Karahashi and Amano reported that LPS can induce signals for the activation of caspase-3-like protease, a key enzyme regulating apoptotic cell damage in a macrophage-like cell line (Karahashi and Amano 1999). In this study, the immunohistochemical localization of caspase-3 appeared strongly expressed, while Bcl-2 appeared weakly expressed in the ovarian tissues of LPS maternally treated offspring. After treatment with Zn-NPs, the expression of caspase-3 declined, while Bcl-2 expression was markedly increased. This finding explains that LPS is implicated in the induction of apoptosis in the ovary of offspring. Similar observations were recorded in the ovarian tissues of bovines treated with LPS (Storni et al. 2022). The apoptotic effects induced in the ovaries of offspring maternally treated by LPS may be attributed to the direct apoptotic effects of LPS on the placenta (Ejima et al. 2000). Kirsten et al. revealed that LPS exposure during gestation is implicated in zinc deficiency (hypozincemia) and consequently decreased zinc supply to the fetuses, resulting in apoptotic effects on the normal growth of the fetuses (Kirsten et al. 2015). In the current work, post-Zn-NP supplementation with LPS during gestation successfully attenuated the apoptotic effects of LPS on the ovarian tissues of offspring. This finding agrees with previous reports (Ota et al. 2015; Wilson et al. 2016) that found that zinc supplementation during gestation is necessary for normal growth and development of gonads of fetuses. Deshpande et al. reported that Zn plays a major role in maternal, infant, and neonatal survival (Deshpande et al. 2013). Also, zinc plays an essential role in the normal development of ovarian embryos during gestation (Ebisch et al. 2007; Cummings and Kovacic 2009). Wilson et al. added that maternal zinc deficiency increases the risk of fetal mortality and growth retardation (Wilson et al. 2016). Additionally, zinc deficiency in women was found to induce several pathological conditions, such as impaired synthesis of FSH and LH, abnormal ovarian development, and gross congenital malformations of fetuses (Cummings and Kovacic 2009; Wang et al. 2015). Also, it has been revealed that supplementation of pregnant women with zinc is associated with normal growth in both term and preterm infants over the first 12 months (Giudice 2010; Sharif et al. 2017). Moreover, Zn deficiency is associated with oxidative stress-driven apoptosis (Roscioli et al. 2013). Caspase-3 and Bcl-2 are important genes and proteins through which a Zn deficiency results in apoptosis (Roscioli et al. 2013). The regulation of apoptosis by Zn via Bcl-2 and caspase-3 plays a major part in cellular protection (Siddiqui et al. 2015). The mechanism by which Zn protects against apoptosis involves inhibition of Ca^2+^ and Mg^2+^-dependent endonuclease action that results in apoptosis (Evgeni et al. 2014). Also, Zn acts as an antioxidant promoter and mediator that engulfs ROS through various means, resulting in antiapoptotic effects on cells (Dunnill et al. 2017). All these previous reports explain the antiapoptotic effects of zinc nanoparticles via regulation of the activity of caspase-3 and Bcl-2 in the ovarian tissues of LPS maternally treated offspring.

Transforming growth factor beta (TGF-β) is a multifunctional cytokine belonging to the transforming growth factor superfamily, which is produced by all white blood cell lineages (Meng et al. 2016). Moreover, the first members of TGF-β superfamily were identified on the basis of their ability to induce a transformed phenotype in certain cells in culture (Lutz and Knaus 2002). They are also known as multifunctional polypeptides involved in the regulation of cell proliferation and differentiation, immune regulation, angiogenesis, and the regulation of extracellular matrix (Rich et al. 2001). Additionally, TGF-β plays an essential role in the induction of apoptosis mediated by caspases in several cell types (Perlman et al. 2001; Herrera et al. 2002). Rosairo et al. reported that TGF-β is necessary for the development of ovarian follicles during pre- and postnatal life (Rosairo et al. 2008). In the current work, a highly significant activity of TGF-β was recorded in the ovarian tissues of LPS maternally treated offspring. However, after treatment with Zn-NPs, the level of TGF-β appeared significantly lower when compared with the control. Sun et al. found overexpression of TGF-β in the pancreatic cells of LPS-treated rats (Sun et al. 2018); the authors explained that overexpression of TGF-β is implicated in the induction of pancreatic cell apoptosis and fibrosis. In our results, overexpression of TGF-β in the ovarian tissues of LPS maternally treated offspring may be a causative factor for degenerative follicles appearing in the ovarian histopathological observations. The role of Zn-NPs in regulating the activity of TGF-β in the ovarian tissues in this study may be attributed to their direct inhibitory effect on the apoptotic pathway.

LPS can stimulate monocyte and macrophage cells to secrete nitric oxide (NO) and inflammatory substances (cytokines), such as tumor necrosis factor-alpha (TNF-α) (Kikkawa et al. 1998). Prasad found a remarkable increase in the level of TNF-α produced by monocytes and macrophages, which were activated by the inflammatory process induced by bacterial LPS and were associated with decreased zinc levels in patients (Prasad 2008). This, in parallel with the findings of this study, showed a significant increase in TNF-α activity in the ovarian tissues of LPS maternally treated offspring compared with the control. Furthermore, LPS from bacteria causes damage to the tissues and promotes the release of thrombin, histamine, and cytokines (Janeway et al. 2005).

Zinc supplementation in LPS-treated mice was shown to alleviate the elevated level of serum TNF-α (Yusuf et al. 2019). Such a finding goes parallel with our obtained result, which showed highly significant decreased activity of TNF-α in the ovarian tissues of LPS + Zn-NPs maternally treated offspring. The potential role of Zn against LPS-caused excessive liberation of TNF-α is mainly attributed to its role in inhibiting the formation of free radicals via increasing the formation of antioxidant enzymes (SOD and CAT). These antioxidants inhibit the release of TNF-α (Rosalina 2007).

## Conclusion

LPS exposure during the early gestation period is implicated in the induction of deleterious biochemical and histological changes in the gonads of mother rats and their female pups. Zn-NPs have a powerful ameliorative role against the most deleterious biochemical and histopathological changes induced in gonads by LPS via activation of antioxidant enzymes and inhibition of the apoptotic effect of LPS.

## Data Availability

The datasets used and/or analyzed during the current study available from the corresponding author on reasonable request.
